# Analysis of three-dimensional chromatin packing domains by chromatin scanning transmission electron microscopy (ChromSTEM)

**DOI:** 10.1038/s41598-022-16028-2

**Published:** 2022-07-16

**Authors:** Yue Li, Vasundhara Agrawal, Ranya K. A. Virk, Eric Roth, Wing Shun Li, Adam Eshein, Jane Frederick, Kai Huang, Luay Almassalha, Reiner Bleher, Marcelo A. Carignano, Igal Szleifer, Vinayak P. Dravid, Vadim Backman

**Affiliations:** 1 Applied Physics Program, Northwestern University, Evanston, IL 60208 USA; 2 Department of Biomedical Engineering, Northwestern University, Evanston, IL 60208 USA; 3 Department of Materials Sciences and Engineering, Northwestern University, Evanston, IL 60208 USA; 4 Shenzhen Bay Laboratory, Institute of Systems and Physical Biology, Shenzhen, 518132 China; 5 Medical Scientist Training Program, Feinberg School of Medicine, Northwestern University, Evanston, IL 60611 USA; 6 Department of Chemistry, Northwestern University, Evanston, IL 60208 USA

**Keywords:** Chromatin, Imaging techniques, Biological physics

## Abstract

Chromatin organization over multiple length scales plays a critical role in the regulation of transcription. Deciphering the interplay of these processes requires high-resolution, three-dimensional, quantitative imaging of chromatin structure in vitro*.* Herein, we introduce ChromSTEM, a method that utilizes high-angle annular dark-field imaging and tomography in scanning transmission electron microscopy combined with DNA-specific staining for electron microscopy. We utilized ChromSTEM for an in-depth quantification of 3D chromatin conformation with high spatial resolution and contrast, allowing for characterization of higher-order chromatin structure almost down to the level of the DNA base pair. Employing mass scaling analysis on ChromSTEM mass density tomograms, we observed that chromatin forms spatially well-defined higher-order domains, around 80 nm in radius. Within domains, chromatin exhibits a polymeric fractal-like behavior and a radially decreasing mass-density from the center to the periphery. Unlike other nanoimaging and analysis techniques, we demonstrate that our unique combination of this high-resolution imaging technique with polymer physics-based analysis enables us to (i) investigate the chromatin conformation within packing domains and (ii) quantify statistical descriptors of chromatin structure that are relevant to transcription. We observe that packing domains have heterogeneous morphological properties even within the same cell line, underlying the potential role of statistical chromatin packing in regulating gene expression within eukaryotic nuclei.

## Introduction

Three-dimensional chromatin packing in the cell nucleus plays an important role in regulating numerous cellular processes, and large-scale alterations in chromatin structure are associated with cancer, neurological and autoimmune disorders, and other complex diseases^1–3^. The fundamental repeating unit of chromatin is the nucleosome, in which 147 bp of the DNA is wrapped around a core histone octamer^4^. The core particle adopts a squat cylindrical shape, with a diameter and height of approximately 11 nm and 5.5 nm, respectively^5^. The nucleosome is the first level of higher-order packing of the chromosomal DNA. Nucleosomes are connected by linker DNA, which altogether forms the 10-nm chromatin fiber^6^. Central to the textbook view of chromatin packing is that 10-nm chromatin fibers assemble into 30-nm fibers, that further fold into 120-nm chromonema, to 300- to 700-nm chromatids, and ultimately, mitotic chromosomes^7–10^.

However, the key tenant of this view, the 30-nm fiber, has been challenged by an abundance of recent evidence. Various studies using cryo-electron microscopy, small-angle X-ray scattering, electron spectroscopy imaging, and super-resolution microscopy failed to observe 30-nm fibers in interphase chromatin or mitotic chromosomes in numerous cell lines^11–14^. For example, Ricci et al. observed the existence of heterogeneous nucleosome ‘clutches’ at the level of the primary fiber, the size of which depends on the epigenetic state and cell type^14^. Recently, a combination of DNA-specific staining (ChromEM) and multi-tilt electron tomography (ChromEMT) observed in situ that the chromatin fiber consists of disordered fibers that have diameters between 5 and 24 nm during both interphase and mitosis, with a higher packing concentration in mitotic chromosomes^15^. Altogether, these studies suggest that the interphase chromatin and mitotic chromosome organization is constructed by 10-nm fibers without folding into 30-nm fibers^12,16,17^. In this new paradigm, the 10-nm fibers condense into highly disordered and interdigitated states, which may be constantly moving and rearranging at the local level^18–20^.

Despite their dynamic and fluid-like nature, several complementary optical imaging and genomics studies have revealed higher-order, domain-like structures above the level of the primary fiber. ‘Chromomeres’, punctate chromatin particles around 200–300 nm in diameter, have been observed in both interphase chromatin and mitotic chromosomes using stimulated emission depletion (STED) microscopy^21^. A recent study employing photoactivated localization microscopy (PALM) live-cell imaging in mammalian cells determined that nucleosomes are arranged into physically compact chromatin domains with a 160 nm diameter^22^. The dynamics of these chromatin domains were correlated with those of replication domains, which range in diameter between 110 and 150 nm^22–25^. In mammalian cells, 3D-structured illumination microscopy (SIM) imaging demonstrated that DNA labeled with fluorescent in situ hybridization (FISH) forms chromatin domain clusters (CDCs) of around 120 to 150 nm in diameter in which the chromatin compaction increases radially from the periphery towards the CDC core^26^. Meanwhile, chromatin conformation capture (3C) and related methods (4C, 5C, Hi-C, Dip-C) have revealed that the eukaryotic genome is partitioned into topologically associating domains (TADs) at the scale of several hundreds of kilobases (kbs) and smaller loop domains, or sub-TADs^27–30^. Recently, high-resolution imaging experiments have visualized TADs identified by Hi-C as compacted domains in single cells, providing a link between the nanoscopic spatial structures and genomic domains^31,32^. Altogether these higher-order chromatin structures potentially play an important role in DNA-based processes, such as transcription, replication, and repair, and perhaps extend to complex processes, such as aging and diseases such as cancer^33–36^.

In parallel with experimental findings, many polymer models have been proposed to understand the basic folding principles of these chromatin domains. For example, the fractal globule model describes chromatin as a collapsed polymer where topological constraints result in a hierarchical organization of non-entangled structures. This model explains earlier Hi-C results but was later challenged by in situ higher resolution data^37,38^. Additionally, chromatin domains observed by recent PALM imaging deviate from the fractal globule model at large length scales^39^. More recently, a logarithmic fractal model was proposed to describe the large-scale organization of chromatin based on small-angle-neutron-scattering (SANS) experiments^40^. Additional statistical models of chromatin have been proposed, including the novel self-returning random walk (SRRW) model, which depicts chromatin as non-globular, porous, and irregular “tree” domains and can reproduce key experimental observations including TAD-like features observed in Hi-C contact maps^41^.

Employing both fixed and live-cell nanoimaging, we have recently uncovered the existence of chromatin packing domains, which have similar statistical packing behavior^42^. Our computational model of transcription predicts that global transcription patterns and the phenotypic plasticity of cells are influenced by macromolecular crowding^43–46^. Crowders are macromolecules that exclude volume and, thus, directly modulate the kinetics and efficiency of chemical reactions such as transcription^47,48^. In the nucleus, chromatin density is the major crowder, and the statistical packing of chromatin within these domains characterizes the distribution of chromatin mass-density, directly influencing gene expression. To properly characterize this statistical packing behavior of domains along with other functionally relevant morphological properties requires an imaging modality that provides structural data with high resolution at the level of the DNA base pair, the functional unit of transcription.

However, the resolution limit of super-resolution microscopy (20–30 nm) and 3C methods (Hi-C ~ 5 kb, Micro-C ~ 200 bp^49^) leave us with a less-than-precise understanding of the folding of DNA (~ 2 nm per bp) into higher-order chromatin structures. Additionally, many computational models are validated or parametrized by either population-level data from genome-wide Hi-C contact maps, which provide a 2D representation of 3D chromatin connectivity, or 3D distances and spatial location of specific genomic loci determined by DNA-labeling with super-resolution microscopy^50–52^. However, neither technique can both characterize 3D chromatin conformation with a micron-scale field of view and provide information down to the level of a single DNA base pair. Thus, a new integrated experimental technique and analysis methodology is required to determine additional model parameters that will provide the most realistic description of the 3D chromatin structure to build more accurate chromatin polymer models. Such a modality would ideally be able to relate experimentally observed structural data with a physics-based framework of gene expression to further characterize the assembly, packaging, and morphology of chromatin domains in situ in the context of transcription.

In this paper, we utilized scanning transmission electron microscopy tomography with ChromEM staining (ChromSTEM) to resolve the 3D chromatin organization for two mammalian cell lines in vitro. ChromSTEM provides visualization of DNA structure down to sub-4 nm-resolution with image intensity linearly related to DNA density. Previous studies employing Click-EM DNA labeling have first binarized EM images, which loses pertinent information^15,42^. Importantly, this is the first study that has analyzed statistical packing behavior without first binarizing. We observed that chromatin fibers fold into distinct, anisotropic packing domains in which the mass scaling behavior within domains follows a near-power-law relationship. We further quantified the physical properties related to transcription processes. These include the chromatin volume concentration (CVC) which is correlated with the transportation and binding efficiency of transcriptional reactants, and the exposure ratio which is related to accessibility of genes within these domains. We find that these transcriptional properties have a direct relationship with the chromatin packing scaling and the size of packing domains, unveiling a potentially important link between the statistical chromatin structure within domains and functionality. Finally, using chromatin transmission electron microscopy with ChromEM labeling (ChromTEM) to extend the statistical yield of ChromSTEM, we estimated the chromatin packing scaling behavior at the peripheral transcriptionally inactive, and the interior chromatin within whole nuclei which further indicate the involvement of such statistical descriptors of chromatin in local transcription.

## Results

### ChromSTEM imaging of chromatin density distribution in mammalian cells

**Figure 1:**
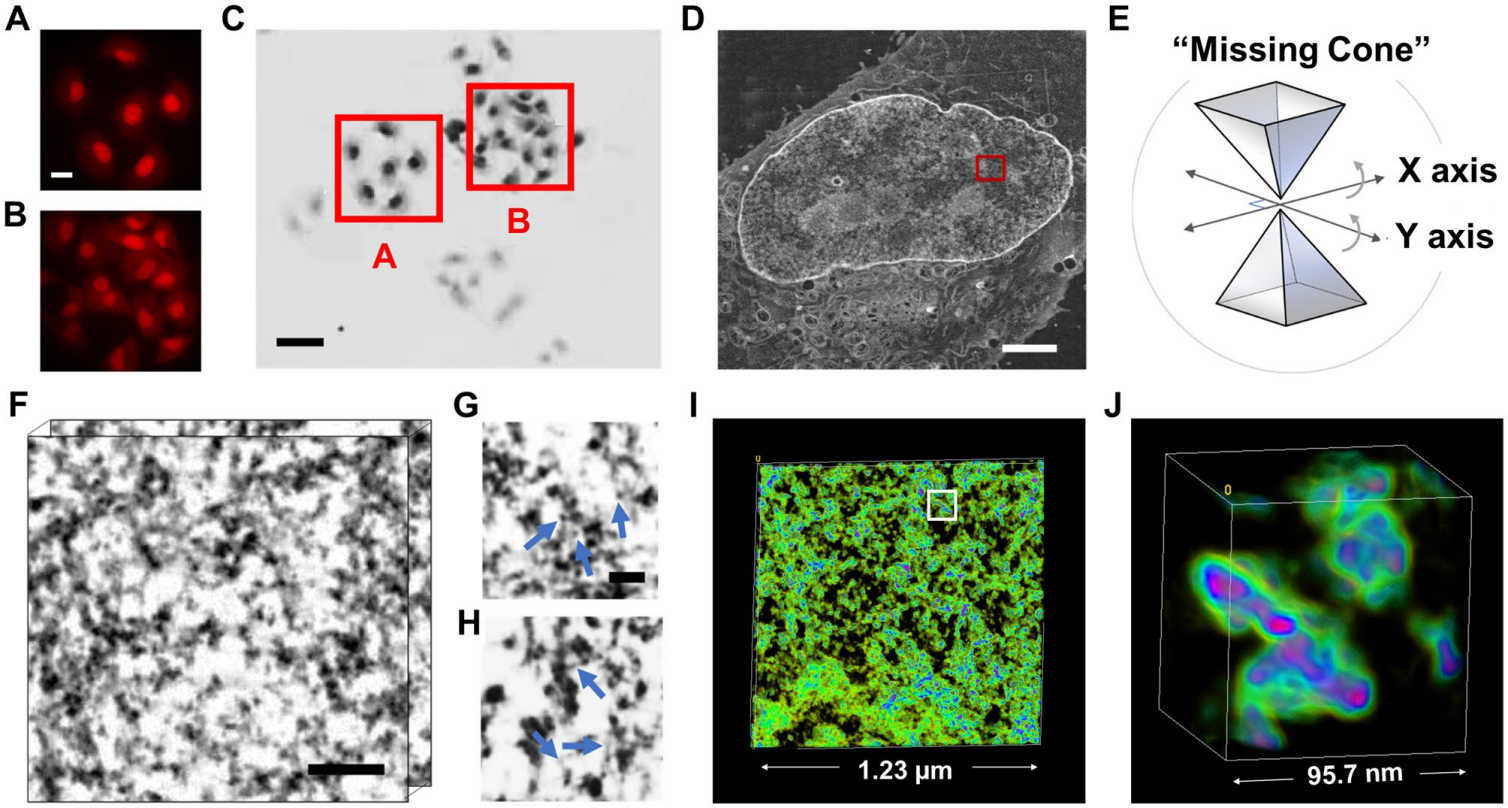
ChromSTEM tomography reconstruction of chromatin in an A549 cell. (**A**, **B**) The DRAQ5 photo-oxidation process takes 7 min for each region of interest. Scale bar: 10 µm. (**C**) The DRAQ5-labeled regions were more intensely stained than the nearby regions (red squares; the letter corresponds to the regions in the left panels). Scale bar: 20 µm. (**D**) STEM image of a 100 nm thick section of an A549 cell in HAADF mode. Scale bar: 2 µm. (**E**) Schematics for dual-tilt tomography. The sample was tilted from − 60° to 60° with 2° increments on two perpendicular axes. (**F**) 3D tomography of the A549 chromatin. Scale bar: 120 nm. (**G**, **H**) The fine structure of the chromatin fiber: nucleosomes (blue arrows in **G**), linker DNA (blue arrows in **H**) Scale bar: 30 nm. (**I**, **J**) 3D rendering of the chromatin organization, the pseudo-color was based on the intensity of the tomograms (Mov. S4–S6). (**I**) A magnified view of the region labeled by a white square in (**J**). Pink and green regions represent high and low DNA density regions, respectively.

Following the ChromEM protocol reported previously^15^, we labeled the DNA of human pulmonary adenocarcinoma epithelial (A549, Fig. 1, Mov. S1) and human fibroblast (BJ, Fig. S1, Mov. S2) cells to characterize the chromatin packing behavior in two genetically distinct cell lines and determine overall generalities. After resin embedding, the labeled regions can be identified based on image contrast in bright field optical micrographs: the photo-oxidized cells appeared significantly darker than the non-photobleached cells (Fig. 1A–C). Dual-tilt STEM tomography in HAADF mode was performed for part of the nucleus (~ 2 μm × 2 μm) on a 100 nm thick resin section. Within the same tomogram, there are large variations in DNA contrast potentially indicating the coexistence of euchromatin and heterochromatin-rich regions (red box in Fig. 1D). Only interior sections of the nucleus were analyzed, as images with peripheral chromatin may also include signal emerging from the nuclear envelope which would need to be accurately excluded from each slice of the 3D tomography reconstruction. Unlike the near-binary image contrast from the conventional EM staining and analysis methods^15^, our ChromSTEM platform provides continuous variations of the DNA contrast inside the nucleus. Each tilt series was aligned with fiducial markers in IMOD^53^ and reconstructed by a penalized maximum likelihood (PLM-hybrid) algorithm in Tomopy^54^. The two sets of tomograms were combined in IMOD to suppress missing wedge (Fig. 1E, S2) artifacts^55^. The final tomograms (Fig. 1F) had voxel sizes varying from 1.8 to 2.9 nm, with clearly resolved nucleosomes (Fig. 1G, S3A) and linker DNA (Fig. 1H, S3B). We also identified rare occurrences of distinct higher-order supranucleosomal structures, such as stacks and rings (Fig. S3C–D). Examples of the full stack of tomography are shown in Mov. S1–S3. The 3D volume of the chromatin was rendered in the FIJI volume viewer^56^ (Fig. 1I–J, Mov. S4–S9). The voxel intensity of the tomogram was used for color-coding as it highlights the continuous variations of chromatin density within the nucleus.

### ChromSTEM reveals chromatin packing domains with similar mass scaling behavior

Due to its semi-flexible nature, the chromatin polymer can, in principle, adopt an infinite number of 3D conformations which are not conserved temporally or across cell populations^57^. However, the statistical properties of the chromatin structure can be characterized. One property used commonly in polymer physics to describe statistical polymer organization is scaling laws, which determine how the number of monomers or, equivalently, the mass of the polymer, scales with the physical space the polymer occupies^58^. The scaling laws of a homopolymer chain, where all monomers interact in the same way, are dependent on the balance of the free energy of polymer–polymer compared to polymer–solvent interactions. Under dilute, equilibrium conditions, such homopolymers are expected to exhibit mass scaling characterized by a power-law relationship between the mass (*M*) and the size *r* at certain length scales: $M \propto {r}^{D}$ , where *D* is the mass scaling coefficient, or the packing scaling, of the polymer. When the interaction of monomers with the solvent is preferred, $5/3$ < *D* < 2. When self-interaction is preferred, $2$ < *D* < 3. In a good solvent, *D* = 5/3 and the polymer adopts a swollen self-avoiding walk. When a polymer’s self-interaction and interaction with the encompassing solvent are equally preferred, as in the case of a random walk, *D* = 2. A special case of *D* = 3 is the collapsed fractal globule structure^37,59^.

Chromatin itself exists as a heteropolymer. Its monomers, i.e., nucleosomes, possess varying biochemical properties in the form of epigenetic modification, such as DNA methylation and post-translational histone modifications. Chromatin conformation can be further influenced by active molecular mechanisms that impose additional topological constraints, including CTCF-cohesin or transcription-dependent looping, interactions with nuclear lamins, and phase separation driven by chromatin-associated proteins such as HP1^60,61^. Therefore, at any given point in time, chromatin conformation is determined by different, and potentially competing forces, such active constraints and the balance between chromatin-chromatin and chromatin-nucleoplasm interactions, altogether resulting in a non-equilibrium system. Additionally, chromatin occupies a significant volume fraction within the nucleus. As a result, the intranuclear chromatin environment is both non-equilibrium and non-dilute, and thus, the rules of polymer physics do not guarantee that the genome-wide structure of chromatin can be described using the same power law-scaling relationship.

This, within the nucleus, there may be separate regimes or length scales in which chromatin exhibits different mass scaling behavior. For example, (1) the primary 10-nm chromatin fiber may exhibit a unique intra-fiber scaling compared to larger length scales, (2) higher-order chromatin domains with power-law packing scaling behavior could exist within certain regimes, and (3) for length scales above the size of the individual domains there could be an additional structured organization of domains or random distribution of spatially uncorrelated domains.

**Figure 2:**
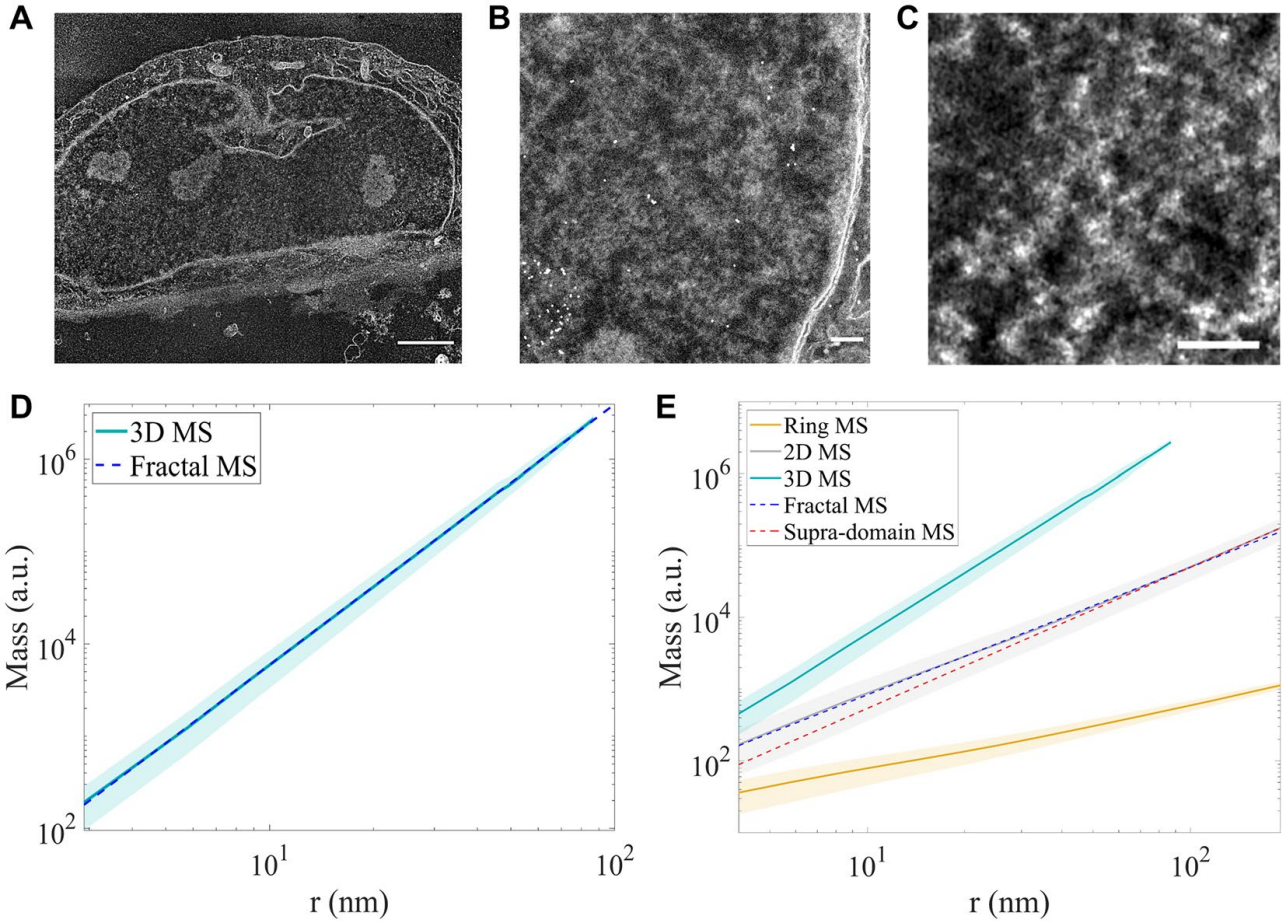
Chromatin folds into packing domains that have similar mass scaling behavior. (**A**) STEM HAADF image of a 150 nm section of a BJ cell nucleus for tomography reconstruction. Scale bar: 2 µm. (**B**) A magnified view of the chromatin and the nuclear periphery of the same cell in (**A**) with gold fiducial markers (white). The intensity variation of the image shows that the chromatin packs at different densities throughout. Scale bar: 200 nm. (**C**) A virtual 2D slice of the chromatin of a BJ cell after tomography reconstruction. Scale bar: 100 nm. The mass scaling analysis was performed on the gray scale tomograms (**D**, **E**) The average mass scaling (MS) curves from the analysis of four A549 cells in (**D**) 3D and (**E**) Ring, 2D, and 3D. The mass scaling was conducted for the entire grayscale tomogram and the average mass scaling curves weighted by the chromatin density values were computed. 3D mass scaling curve exhibits power-law behavior with a single scaling exponent up to r = 60 nm. Two regimes of mass scaling with different packing scaling D can be identified. In the 2D cases, the MS curve starts with a packing scaling with D_log_ < 3 (blue dashed line) and smoothly transitions to values close to D_log_ = 3 (red dashed line).

To elucidate the chromatin structure within the cell nucleus, we investigated the mass scaling behavior of the continuous signal of DNA contrast obtained from ChromSTEM imaging (Fig. 2A–C). Image acquisition was performed as follows. To first locate the cell of interest, we collected an image of the nucleus at low magnification (~ 2 kX). Next, we collected the tilt series ChromSTEM images in the HAADF mode by selecting a random intranuclear region of interest at high magnification (~ 90 kX) that is located away from the nucleoli and nuclear boundary, or cytoplasm (Fig. 1D,F, 2A–C). The resultant 3D tomogram has high contrast and continuous signal emerging specifically from chromatin. In the analysis, we treat the heterogeneous chromatin fibers, as reported by Ou et al. using ChromEMT, to be the fundamental element in building higher-order structures^15^. Practically, the 3D mass scaling relationship is defined as how the total amount of chromatin (*M*) enclosed within a volume ($V=\frac{4}{3}\pi {r}^{3}$) changes with its radius *r*. The 2D case can be described as a cross-sectional slice of the 3D system. In this case, *M* is the amount of chromatin enclosed within an area ($A=\pi {r}^{2}$). The derivative of the area results in the perimeter, which represents the 1D case. Therefore, in the 1D scenario, *M* is the amount of chromatin positioned on the circumference of a circle ($P=2\pi r$), which we refer to as the 1D case as “ring mass scaling”. We calculated the ring, 2D, and 3D mass scaling by performing linear regression analysis on the log–log mass scaling curves for the given dimensions. The law of additivity of fractal codimensions approximates the conversion of chromatin packing scaling between different dimensions^62^, and we confirmed from our calculations that the 3D mass scaling exponent can be estimated using the 2D and ring mass scaling (Supplementary Methods, Fig. S4A).

As ChromSTEM only provides a snapshot of the chromatin conformation at a single time point, we randomly sampled different regions within the field of view and calculated the mean mass scaling to capture the statistical behavior. We performed mass scaling analysis on tomograms from both A549 and BJ cells. For four A549 cells with a total volume of 1.16 µm^3^ resolved at a voxel size of 2.0 to 2.9 nm, we obtained the mass scaling curves for all three dimensions (Fig. 2D,E, S4B). A total volume of 0.09 µm^3^ was reconstructed from three BJ cells at a voxel size of 1.8 to 2 nm and mass scaling analysis was performed (Fig. S4C). To identify length scales where a single packing scaling exponent cannot sufficiently describe the mass scaling behavior, and to determine average packing scaling within this regime, we evaluated the derivative of the log–log scale of the 3D and 2D mass scaling curves as a function of *r*. The slope, *D*_*log*_ was defined as a linear regression fit to the log–log scale of the mass scaling curves. In this linear regression fit, *D*_*log*_ should be equivalent to the packing scaling, *D*, within the power-law scaling regime. Power-law scaling occurs when the length scales associated with *D*_*log*_ extend over at least one order of magnitude. From our 3D mass scaling analysis on A549 cells, we observed a power-law mass scaling regime extending from 2 to 60 nm with a fitting parameter of *D*_*log*_ = 2.82 ± 0.01 (Fig. 2D, blue dashed line). We refer to the region where power-law mass scaling occurs with one chromatin packing scaling exponent as the packing domain regime. From ~ 60 to 90 nm, a gradual increase in *D*_*log*_ to about 2.92 ± 0.02 was observed, which we refer to as the supra-domain regime. However, because the maximum section thickness of A549 tomograms was 180 nm, our 3D analysis was unable to reliably evaluate mass scaling behavior above 90 nm. Additionally, we did not perform the 3D mass scaling analysis for BJ cells, as the thickness of the reconstructed section of BJ cells was smaller than 70 nm, and the 3D mass scaling curve would only extend up to 35 nm.

Due to the intrinsic length-scale limitation of 3D mass scaling determined by section thickness, we next performed the mass scaling analysis at different dimensions for both A549 and BJ cells. Employing the law of additivity of fractal codimensions, we calculated the 3D mass scaling exponent from 2D and 1D mass scaling curves as: ${D}_{3D}={D}_{2D}+1$, and ${D}_{3D}={D}_{1D}+2$ (Fig. S4B)^62^. For both A549 and BJ cells, we first evaluated the slope of the 2D mass scaling curve in the log–log scale along its entire length using a 12 nm sliding window. By estimating the local slope for small ranges of *r* along the entire length of the 2D mass scaling curves, two distinct regimes were identified. The first regime extended up to *r* ~ 55 nm, followed by a gradual increase in the local log–log derivative towards a value of *D*_*log*_ ~ 3. Similar to the 3D mass scaling analysis, for A549 cells (Fig. 2E), we then obtained the slope of linear regression, *D*_*log*_ $=$ 2.74 ± 0.01 for 2 nm < *r* < 55 nm (blue dashed line). Above these length scales (r ~ 60 nm), the slope continuously increases until it approaches 3 for *r* > 145 nm (red dashed line) up to 200 nm. Similarly, for BJ cells (Fig. S4C), the fitting parameter for the linear regression was estimated to be *D*_*log*_ $=2.78 \pm 0.01$ (blue dashed line) for 2 nm < *r* < 50 nm and *D*_*log*_ approaches 3 (red dashed line) for *r* > 100 nm. The shift from the domain regime with similar packing scaling behavior (2 nm < *r* < 50–60 nm) to the supra-domain regime where *D*_*log*_ ~ 3 (*r* > 100–145 nm) is continuous, as opposed to a sharp, biphasic transition. The implications of this result will be discussed later.

Besides the two regimes determined by 2D and 3D mass scaling, the ring mass scaling curve exhibits a third regime from 2 nm < *r* < 10 nm for both cell lines (Fig. S4D,E). This can be interpreted as the chromatin fiber regime. The upper length scale of 10 nm agrees with the upper limit of the primary chromatin fiber size (24 nm maximum diameter)^15^. However, this regime was not identified on the mass scaling curves of higher dimensions, possibly due to limited tomography resolution.

Therefore, both the average 3D and 2D mass scaling analyses suggest that for length scales up to 60 nm, chromatin packs into domains that statistically exhibit internal mass scaling behavior and can potentially be described by one average packing scaling exponent. From 2D mass scaling analyses, at larger supra-domain length scales, a gradual increase in D towards a value of 3 was observed. Furthermore, to perform a comprehensive analysis of the 3D chromatin conformation, we then evaluated the radial density and mass scaling profile of individual domains as a function of distance from the center of the domains.

### Quantifying domain size and chromatin packing behavior at the domain boundary

**Figure 3:**
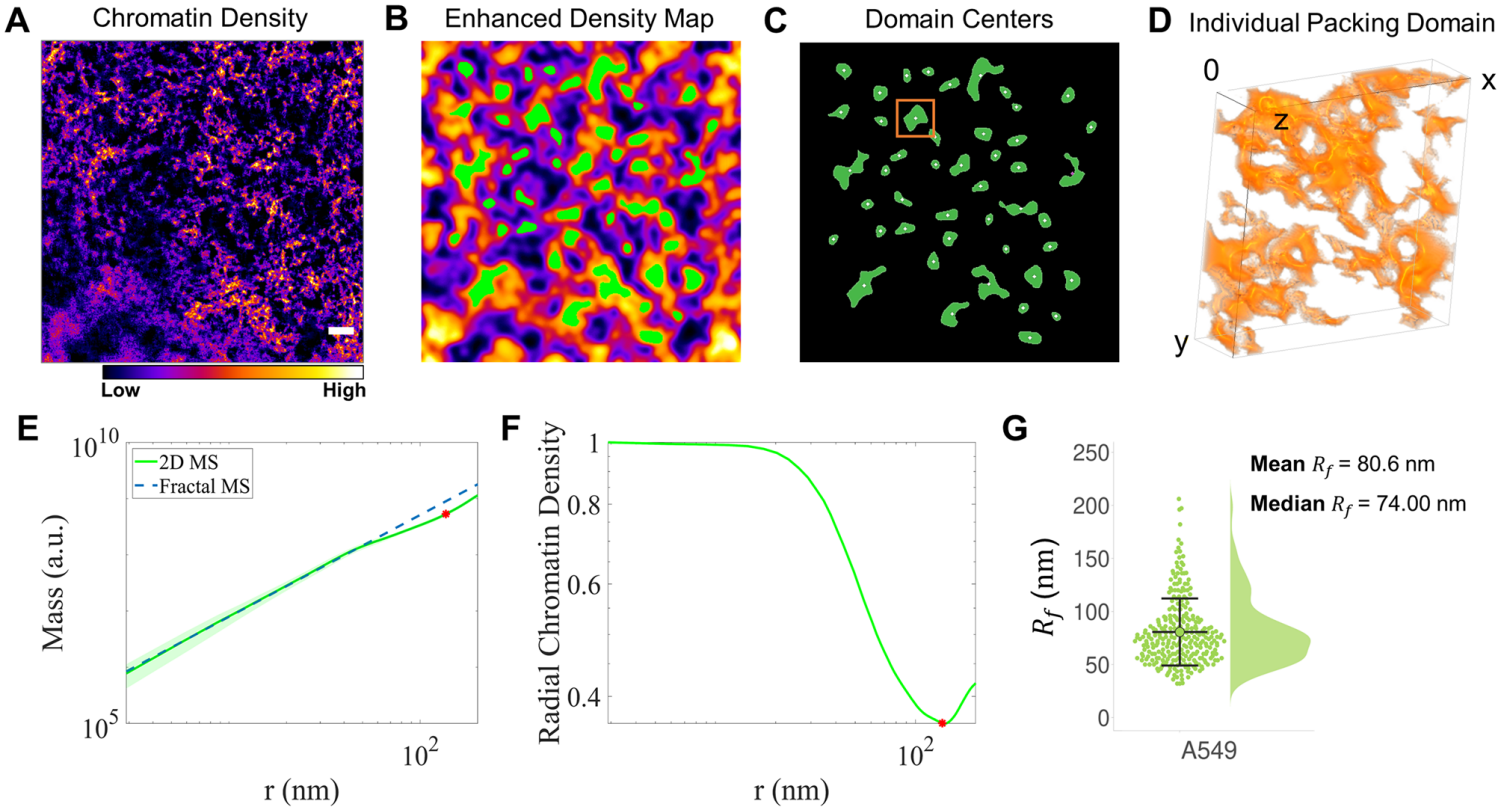
Quantifying domain size and chromatin packing behavior at the domain boundaries. Chromatin packing domains are structurally heterogeneous and anisotropic. (**A**) ChromSTEM grayscale tomogram for one field of view of an A549 cell. The color bar represents chromatin mass density. (**B**, **C**) Local chromatin maxima map estimated from enhanced chromatin density projection shown in (**B**) was utilized to find chromatin domain centers shown in (**C**). (**D**) 3D rendering of the surface of chromatin density in a region containing the packing domain of interest (orange square in **C**). (E) The average 2D mass scaling curve of the chromatin domain within the region of interest (orange square in **C**). For one domain, the mass scaling curve is resampled from all loci within the domain center identified in (**C**). The mass scaling analysis is conducted starting from the domain center. The MS curve starts with D < 3 (blue dashed line) and transitions to values closer to D = 3 beyond the potential domain boundary (red asterisk at 110 nm). (**F**) Radial distribution of chromatin density or radial CVC for the same domain. The radial CVC initially decreases slowly within the domain regime. As the length scale approaches the potential domain boundary (red asterisk at 130 nm), the radial CVC rapidly dips which is followed by a recovery, due to the presence of other domains at those length scales. (**G**) The distribution of R_f_, the radius of packing domains, for A549 cells. The mean R_f_ is 80.6 nm, and the median along with the lower and upper quartile is 74 (58–93) nm.

Our previous analysis averaged mass scaling behavior from all domains analyzed within a given field of view. Next, we wanted to better characterize the mass scaling behavior of individual domains. Here, we outline the criteria to define packing domain boundaries, which involves analyzing both mass scaling behavior and radial chromatin density. First, we identify chromatin domain centers using grayscale ChromSTEM z-stacks with local chromatin density information (Fig. 3A–C, Fig. S5). We interpret length scales in our single domain mass scaling analysis as the physical distance from these domain centers. Within this “center region”, chromatin has similar statistical mass scaling behavior. The boundary of this scaling behavior, equivalent to the size of the packing domain, can be defined as the length scale where the chromatin packing scaling, which defines the mass scaling behavior of the domain, deviates significantly from the statistical behavior of the chromatin within the “center region”. At the same time, for an isolated domain with *D* < 3, the chromatin density decreases from the “center region” to the periphery. For spatially separable domains which exhibit distinct mass scaling behavior, the radial chromatin density per domain is expected to initially decrease, followed by an increase due to the intersection with other domains. Thus, the boundary of a single domain can also be dictated by the radial chromatin density profile as the domain boundary is expected to be located at the minima of this density profile.

To begin this more detailed analysis, we first identified the “domain center region” of each packing domain. From the spatial distribution of 3D chromatin density distribution (Fig. 3A, S7A), we applied Gaussian filtering and local contrast enhancement to create a map of local maxima from the projection images with enhanced chromatin contrast (green areas in Fig. 3B). The centers of these local chromatin intensity maps were then identified (Fig. 3C). For each domain, we resampled the mass scaling curves with centers inside the “domain center region” (Fig. 3D, S7B) and determined mass scaling behavior from these “domain centers” up to *r* = 400 nm for A549 cells and *r* = 200 nm for BJ cells due to differences in section thickness between samples. For each individual domain, the average mass scaling curve exhibits a power-law scaling regime with similar chromatin packing scaling and, at larger length scales, a gradual deviation from the initial power-law behavior (Fig. 3E). We performed linear regression on the 2D mass scaling curve and obtained a slope, ${D}_{log}=2.56\pm 0.02$ for *r* up to 100 nm (Fig. 3E, blue dashed line). This power-law scaling relationship can model the mass scaling curve with less than 5% error within the given fitting range, while a more significant divergence is observed beyond *r* = 110 nm (Fig. 3E, red asterisk). Therefore, the mass scaling behavior for a single packing domain demonstrates that the smaller length scales have a packing scaling *D* < 3 and that, as r increases up to around 100 nm, there is a sharp transition to the supra-domain regime with ${D}_{log}$ = 3.

Additionally, we determined the radial distribution of chromatin density, or chromatin volume concentration (CVC), to characterize the changes in chromatin packing density from the “domain center region” to the periphery of individual domains (Fig. 3F). We observed three key trends in the radial CVC at different distances from the domain center: (1) a relatively flat, slowly decreasing curve near the domain center, (2) a rapidly decreasing curve at a moderate distance from the domain center, and (3) an increasing curve at even larger distances. This third trend is likely caused by the inclusion of chromatin from other nearby domains. The transition point from rapid decrease to increase in radial CVC (red asterisk in Fig. 3F) is consistent with the transition point in mass scaling from similar mass scaling behavior to ${D}_{log}$=3 (red asterisk in Fig. 3E), and both are indicative of the edge of the analyzed domain.

For each domain, we quantified the regime of similar mass scaling behavior, or the radius of the packing domain (*R*_*f*_), as the smallest length scale that satisfies the following criteria (Fig. S6A–C): (1) Mass scaling curve deviates from the initial power-law calculated from small length scales by 5%, suggesting a significantly different packing behavior; (2) Local packing scaling *D* reaches 3, highlighting the supra-domain regime; (3) The radial chromatin density begins to increase. We observed a broad range of *R*_*f*_ for both A549 cells with mean $\sqrt{{\langle {R}_{f}\rangle }^{2}}$ = 86.58 nm and BJ cells with $\sqrt{{\langle {R}_{f}\rangle }^{2}}$ = 58.98 nm (Fig. 3G, S6D). Employing our approach from Li et al., the median genomic size of A549 and BJ packing domains are estimated to be 207 kilo-base pairs (kbp) and 82 kbp respectively^42^. As the mass scaling analysis for individual domains reveals two distinct regimes and the distribution of packing domain sizes is heterogeneous, we can interpret that the intermediate regime for our average mass scaling analysis of the entire tomogram may be a result of averaging domains with different sizes (Fig. 2D,E).

We interpreted R_f_ as the length scale where the chromatin mass scaling no longer follows a power-law relationship, or where a single packing scaling is not sufficient to explain the packing behavior. However, this view does not indicate each domain is spherical with radius *R*_*f*_*.* We further quantified the shape of the domain boundary by calculating the 2D asphericity (*A*_*s*_) of the chromatin enclosed by the domain boundary^63,64^. Considering a 2-dimensional ellipse, ${A}_{s}=\frac{{({a}^{2}-{b}^{2})}^{2}}{{({a}^{2}+{b}^{2})}^{2}}$ , where $a$ and $b$ are the semi axes of the ellipse. Here, *A*_*s*_ can take on values from 0 to 1, depending on the ratio $\frac{a}{b}$. For the case $a=b$, *A*_*s*_ = 0 indicates an isotropic or spherical configuration. In the limit, $a\gg b$, *A*_*s*_ = 1 indicates a linear or stretched configuration. To avoid edge effects, we only considered domains that are entirely within the field of view. We estimated the average of *A*_*s*_ to be $0.446 \pm 0.04$ from 280 domains for A549 cells and $0.458 \pm 0.05$ from 104 domains for BJ cells, respectively (Fig. S6E). Altogether, analysis of individual domains from two different cell lines demonstrates that chromatin fibers are packed into anisotropic domains of variable sizes.

### Heterogeneous morphological properties of chromatin packing domains

Statistical descriptors of packing domains, including chromatin packing scaling, average chromatin density, and size of domains were previously determined to be physical regulators of transcription through crowding-mediated effects^46^. In the context of chemical reactions, macromolecular crowders are any protein, nucleotide, or other macromolecules that occupies physical space but does not directly participate in the reaction^47,48^. As transcription reactions are chemical reactions, crowding directly influences both the kinetics and efficiency of transcription^45^. We have previously developed a computational model of transcription in a realistic chromatin environment by considering chromatin density as the major crowder in the nucleus^43,46^. Thus, characterizing the distribution of statistical properties which control chromatin density distribution can help decode the complex chromatin structure–function relationship.

**Figure 4:**
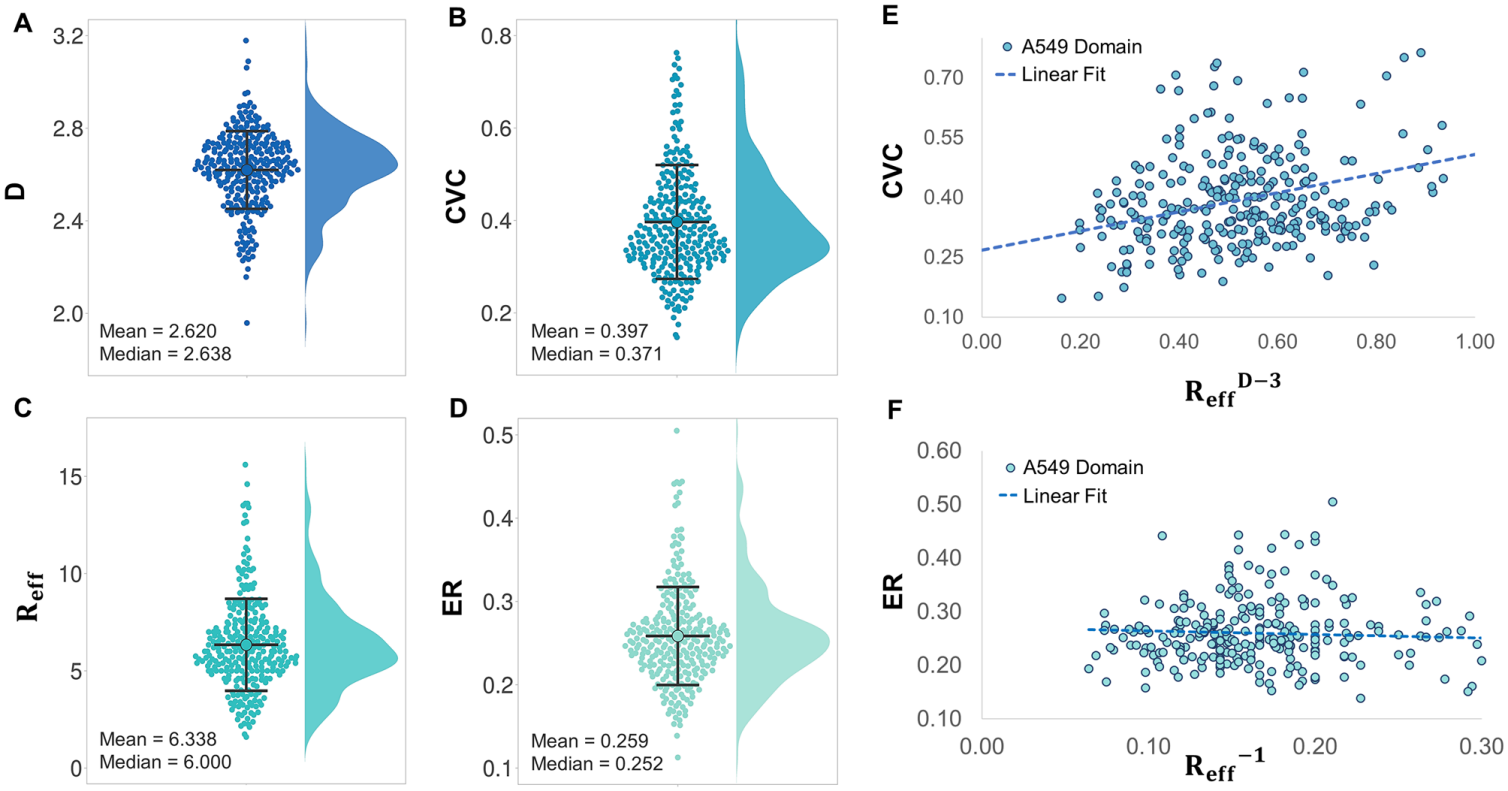
Characterizing morphological properties of chromatin packing domains in A549 cells. A total of 280 A549 cell packing domains were analyzed (**A**) Chromatin packing scaling D distribution was calculated for A549 cells. The median of the wide distribution is equal to 2.64. (**B**) Chromatin volume concentration (CVC) distribution per packing domain. We observed the CVC distribution ranges from 0.15 to 0.92 with a median value of 0.37 for A549 cells. (**C**) Effective domain size R_eff_ for A549 cells. The effective domain size is the ratio between domain size R_f_ and domain fiber size R_min_. For A549 domains, the median R_min_ (IQR) is 11.6 (10–14) nm. (**D**) Exposure Ratio (ER) is defined as the fraction of chromatin voxels on the surface of the interchromatin voids compared to the entire volume. For A549 domains, the median ER ranges from 0.11 to 0.50 with a median value of 0.25. (**E**) A moderate correlation between domain CVC and D has been observed for A549 cells, with r = 0.3. (**F**) Exposure ratio is negatively and weakly correlated with inverse effective domain size for A549, with r = 0.1.

First, for individual domains, we obtained the distribution of packing scaling *D*, with a median and interquartile ranges (IQR) of 2.64 (2.53–2.73) for A549 cells (Fig. 4A), and 2.63 (2.53–2.71) for BJ cells (Fig. S7A–C), both with relatively wide and similar distributions. For the same domains, we determined the average CVC, or chromatin volume concentration, per domain to quantify chromatin compaction. For each pixel, a CVC = 0 signifies there is no chromatin density within the pixel and a CVC = 1 signifies that the entire pixel volume is filled by chromatin density. Similar to the anisotropy analysis, we excluded the domains at the edge of the field of view. We obtained a median (IQR) CVC of 37% (32–45%) for A549 cells (Fig. 4B), and 34% (24–61%) for BJ cells (Fig. S7D), again with large heterogeneity between domains within the same cell line.

For a polymer that exhibits power-law mass scaling behavior within a certain regime, such as chromatin within packing domains, the relationship between mass density, or CVC, and packing scaling follows the relationship ${CVC= \frac{{N}_{f}{V}_{pix}}{{V}_{f}}=A\left(\frac{{R}_{f}}{{R}_{min}}\right)}^{D-3}\propto A{R}_{eff}^{D-3}$. Here, the total mass of chromatin within a domain ${N}_{f}{V}_{pix}=A{\left(\frac{{R}_{f}}{{R}_{min}}\right)}^{D}$ is measured as the product of the number of pixels within the domain that contain chromatin, ${N}_{f}$, and the resolution or smallest unit of chromatin measured by ChromSTEM, ${V}_{pix}$. *R*_*f*_ and ${V}_{f}$ are the domain size and total volume of all pixels within the domain, *R*_*min*_ is the radius of the elementary unit of the chromatin fiber, and ${R}_{eff}=\frac{{R}_{f}}{{R}_{min}}$ is the effective domain size (Fig. 4C)^42^. $A$ is the packing efficiency factor of the fundamental chromatin fiber within the domain. A chromatin domain with $A=1$ specifies that each concentric layer of the domain is packed in the most efficient manner and the mass-density distribution of chromatin is fully designated by the domain packing scaling. Here, we assume that the packing efficiency within the chromatin fiber, the primary building block is 1. In other words, the entire volume of the fiber is completely filled by chromatin. Similar to *R*_*f*_, *R*_*min*_ can be estimated from the limits of the first regime of the ring mass scaling curve. We define *R*_*min*_ as the upper bound of the chromatin fiber regime or the spatial separation that significantly deviates from the mass scaling behavior within the chromatin fiber (Supplementary Methods). Next, we investigated the relationship between average density (CVC), effective size (${R}_{eff}^{D-3}$), and chromatin packing scaling (*D*) within domains across a population of isogenic cells to determine whether there was a universal relationship between these two properties which could be described by one packing efficiency factor and one fiber size. In general, we observed a positive correlation (*r* = 0.3) between CVC and *D* for A549 cells (Fig. 4E). From the ring scaling analysis, we also observed that R_min_ was not significantly different across domains, between cells within the same cell lines, and even between the two cell lines (data not shown). This relationship suggests that the chromatin fiber size may be constant, even across genetically different cells, although the packing efficiency is domain-specific as there is a wide spread of CVC-D relationships that cannot be described by just one packing efficiency factor, *A*, from one linear regression fit (Fig. 4E).

As the boundaries of TADs and chromatin domains are enriched in active transcription processes^42,65^, we next studied how the probability of chromatin being exposed on the surface of domains changes across domains. Here, we define an exposure ratio (*ER*) as the fraction of ChromSTEM voxels on the surface of the domain compared to the total number of pixels encompassing the domain volume. The surface here exclusively refers to the internal surface created by the interchromatin voids within domains. This metric evaluates the surface area to volume ratio of a domain. Without changing the genomic size of a domain, an increase in $ER$ for a given chromatin domain would indicate an increase in the chromatin domain surface, which could increase the amount of surface chromatin that is accessible to transcription processes. First, we define ${A}_{sp}$ as the surface packing efficiency, i.e. the prefactor in the scaling relationship ${S}_{f}={A}_{sp}{S}_{min}{\left(\frac{{R}_{f}}{{R}_{min}}\right)}^{D-1}$ where ${S}_{f}$ is the total surface area of the domain and *S*_*min*_ is the surface area of the elementary unit of the chromatin fiber, measured as the number of pixels. For each domain, *ER* can then be estimated by the following relation: $ER=\frac{\frac{{S}_{f}}{{S}_{min}}}{\frac{{M}_{f}}{{M}_{min}}}={{A}_{ER}{R}_{eff}}^{-1}$, where ${M}_{f}$ is the total mass of a domain, ${M}_{min}$ is the mass of the elementary unit of the chromatin fiber, and ${A}_{ER}=\frac{{A}_{sp}}{A}$ is the exposure ratio efficiency factor and represents the ratio between the packing efficiency at the domain surface, ${A}_{sp}$, compared to throughout the entire domain, $A$. There is a relatively large variability in the distributions of effective domain size, ${R}_{eff}$, (Fig. 4C, S7E) and the exposure ratios (ERs) of domains within each cell line (Fig. 4D, S7F).

Next, we investigated if ${A}_{ER}$ is constant for all domains within the same cell line for both A549 and BJ cells. We performed linear regression analysis to better characterize the relationship between the inverse effective domain size and *ER* at the domain level. We observed a weak negative association (*r* =  − 0.1) between the *ER* and ${{R}_{eff}}^{-1}$ for A549 cells (Fig. 4F). This suggests that the exposure ratio is very weakly dependent on effective domain size and that the exposure ratio efficiency factor is very small, although it varies between domains. Altogether, these results demonstrate that domains have unique morphological properties that are transcriptionally relevant, including average density, packing scaling, packing efficiency, and exposure ratios, that are heterogeneous within the same cell line.

### Distinct chromatin packing behavior for peripheral versus non-peripheral chromatin

Compared to the nuclear interior, chromatin organization at the nuclear periphery presumably has higher density and CVC^15^, is enriched with markers specific to heterochromatin, and demonstrates reduced domain dynamics^22^. Thus, we next wanted to quantify how the chromatin conformation differs between chromatin domains at the nuclear periphery and non-periphery regions by comparing *D* determined from the domain regime between these two regions. To accomplish this, we utilized ChromTEM which enables us to analyze the spatial chromatin density variations within whole nuclei due to its larger field of view^42^. The pseudo-2D quantification of chromatin packing scaling using ChromTEM, allows us to capture the statistical behavior of chromatin from multiple whole nuclei, thus extending the yield of ChromSTEM.

**Figure 5:**
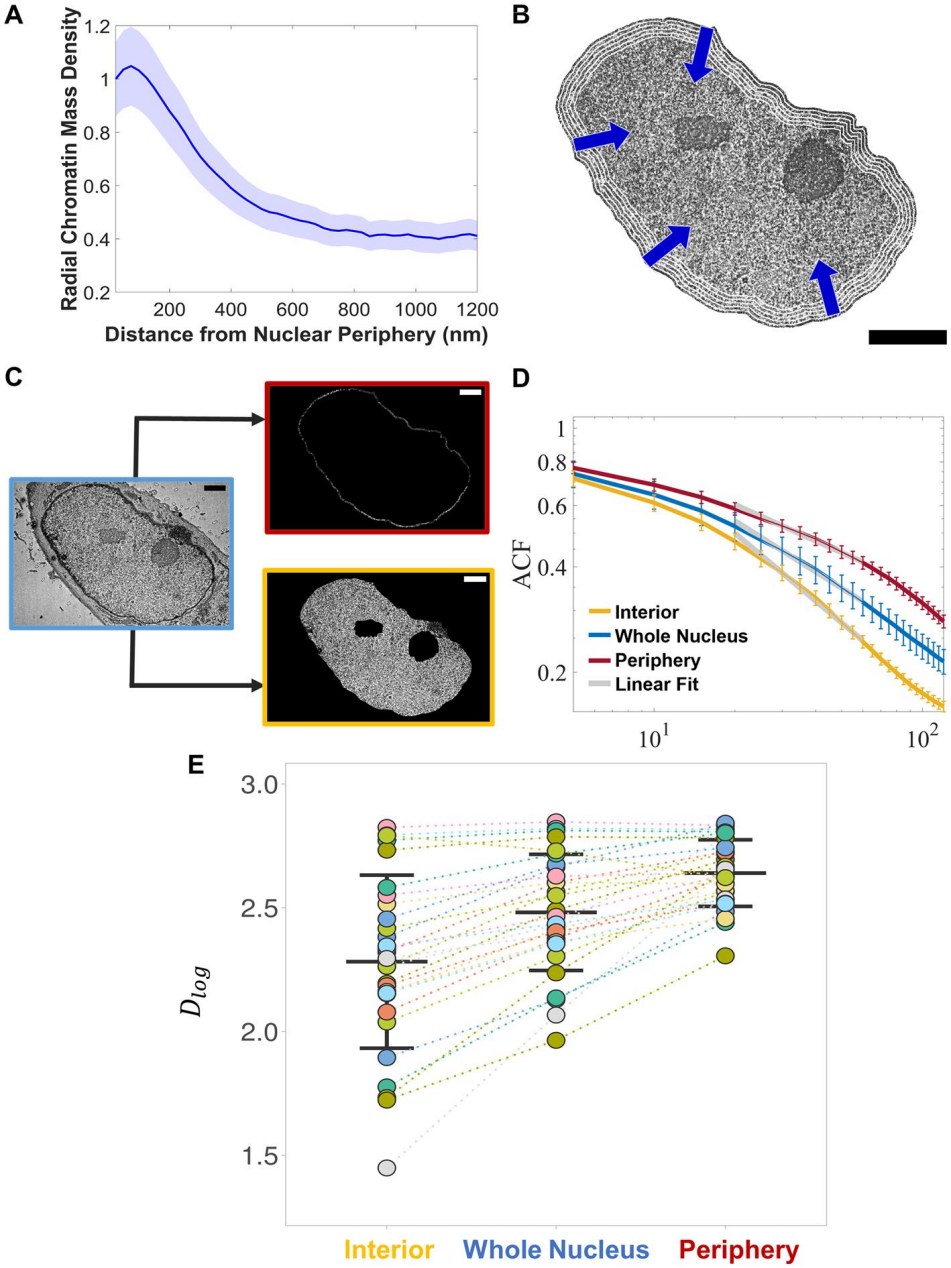
Comparing chromatin organization at nuclear periphery and interior. (**A**) Radial chromatin density was determined using ChromTEM images as a function of distance from the nuclear periphery. (**B**) Illustration showing how the mean radial chromatin mass density in (**A**) was estimated within 25 nm bands from the nuclear periphery to the center. Scale bar, 2 μm (**C**) ChromTEM image of the whole nucleus was utilized to segment chromatin at the nuclear periphery (red), and the interior (yellow). (**D**) The average ACF of chromatin mass density for the whole nucleus (blue), periphery (red), and interior (yellow) in the log–log scale. D was measured inside the domain regime (25 to 60 nm) by a linear regression fit of the ACF on a log–log scale. (**E**) A significantly higher D in the periphery compared to interior regions and the whole nucleus was observed. Each color represents a BJ cell nucleus (N = 29 cells, *p* value < 0.0001). The median D_log_ (IQR) for the nuclear interior is 2.30 (2.11–2.53), the whole nucleus is 2.46 (2.35–2.67), and that for the periphery is 2.64 (2.53–2.76).

We performed ChromTEM imaging of 29 BJ cell nuclei on a 50-nm resin section prepared by ChromEM staining. First, we confirmed that chromatin density is indeed higher at the nuclear periphery and decreases radially as a function of distance from the nuclear periphery, (Fig. 5A,B). We then estimated *D*_*log*_ using the autocorrelation function of spatial variations in chromatin density, ACF(r). As described previously, the ACF of chromatin can be approximated from its mass scaling relationship with respect to length, *r* as $ACF\left(r\right)\propto \frac{dM}{dV}\propto {r}^{D-3}$, and this quantification of *D*_*log*_ is more accurate than mass scaling analysis for the thinner ChromTEM sections^42^. Performing linear regression in the log–log scale on the ACF(r) for the whole nucleus, the nuclear periphery, and the nuclear interior, *D*_*log*_ was estimated for 25 nm < *r* < 60 nm (Fig. 5C,D), which is within the domain regime determined by our ChromSTEM mass scaling analysis. We found that for chromatin at the nuclear periphery, the median *D*_*log*_ (IQR) of 2.64 (2.53–2.76) was significantly higher than that at the nuclear interior, median *D*_*log*_ (IQR) of 2.30 (2.11–2.53) with a *p-value* < 0.0001 (Fig. 5E). Additionally, a wider range of *D* values for the nuclear interior was observed compared to periphery regions. Furthermore, a pairwise comparison of *D*_*log*_ for the whole nucleus, periphery, and interior for each of the 29 analyzed nuclei confirmed that for the majority of the cells the *D*_*log*_ at the periphery was again higher than *D*_*log*_ at the non-periphery (Fig. 5E). These results indicate that in a non-cancer cell with a conventional higher mass-density of chromatin at the nuclear periphery, *D* is drastically different for high-density chromatin associated with LADs than the comparatively less dense chromatin in the interior of the nucleus. Such significant differences between the chromatin at the nuclear periphery which are presumably heterochromatin-rich and the interior which is typically a mix of transcriptionally active euchromatin-and heterochromatin rich regions indicate a potential functional relationship between statistical descriptors of chromatin domains, such as packing scaling and average mass density, and transcription at these regions.

## Discussion

Utilizing ChromEM staining that selectively enhances the contrast of DNA and dual-axis electron tomography with high-angle annular dark-field imaging mode, ChromSTEM has the advantage of resolving chromatin packing in 3D with high spatial resolution at the single-cell level (Fig. 1). Employing ChromSTEM on two genetically different cell lines, both chemically fixed A549 cells (cancer) and BJ cells (non-cancer), we were able to quantify chromatin packing in vitro down to the level of the primary chromatin fiber. Importantly, we studied these cell lines to distinguish basic principles behind chromatin packing that are generally cell line-invariant. We do not assume that the exact results from A549 cells extend to all cancerous cells and that the results obtained from BJ cells represent all non-cancerous cells. By analyzing the mass-scaling behavior of the chromatin polymer, we observed spatially separable and geometrically anisotropic packing domains ~ 80 nm in radius averaging both cell lines (Fig. 3).

The mass scaling within the packing domains follows a power-law relationship with *D* < 3, indicating that chromatin packs into higher-order domains with similar mass-scaling behavior, and that packing domains have radially arranged layers with decreasing chromatin density from the domain center to the periphery. This “core–shell” structure supports earlier experimental work using super-resolution microscopy at a coarser spatial resolution^26,66^. At the same time, the domains are not completely isolated from each other without any chromatin density in between, as CVC values are always above 0. From these observations, it is reasonable to suggest chromatin is organized into complex, porous packing domain structures which are connected by less dense chromatin fibers. The porosity of domains could provide additional surface area, potentially promoting diffusion and targeted search mechanisms, such as transcription. Outside of packing domains, the packing scaling increases to 3 after crossing the domain boundary. A packing scaling of *D*_*log*_ = 3 potentially indicates a random distribution of multiple domains with respect to each other and, importantly, does not substantiate the existence of higher-order packing structures above the level of domains.

Interestingly, the previous ChromEMT study did not observe any higher-order chromatin structures above the level of the primary fiber^15^, which is incongruous with other EM and optical microscopy studies. The size of the packing domains observed using ChromSTEM (~ 160 nm diameter) is consistent with previous observations of higher-order chromatin domains, including ‘chromomeres’ (~ 200–300 nm)^21^, replication domains (~ 110–160 nm)^22–25^, and domains associated with TADs (~ 200–300 nm)^67^. However, the packing domain structures observed using ChromSTEM are obtained at a much higher resolution than previous optical techniques. Additionally, instead of utilizing conventional TEM tomography as reported in the ChromEMT approach^15^, ChromSTEM utilizes quantitative STEM HAADF imaging. Unlike TEM signal, STEM HAADF signal is approximately linearly proportional to the chromatin concentration and therefore enables a more in-depth characterization of higher-order chromatin structures with a sub-2-nm voxel size and ~ 4 nm resolution in single cells. This allows for highly accurate characterization of the packing of the primary chromatin fiber.

Additionally, previous imaging studies have studied domains by either labeling specific genomic regions, including replication domains^22,23,25^ and Hi-C-identified TADs^31,67^ or by delineating boundaries based on absolute chromatin density distribution or coherent motion^21,22,66,67^. On the other hand, ChromSTEM packing domains were determined by a polymer physics-based mass scaling analysis^42^. Thus, the boundaries were (1) not known a priori as in the other labeling experiments, and (2) the statistical packing behavior of these domains has more direct functional implications due to the phenomenon of macromolecular crowding than domains distinguished by chromatin density or motion^46^. Additionally, the high-resolution ChromSTEM technique enables the quantification of chromatin structure down to the level of the DNA base pair, resulting in the identification of packing domains and the analysis of chromatin conformation within these domains, thus providing us with the true 3D chromatin architecture. Functionally important properties of the packing domains, including CVC, domain size, packing scaling, surface exposure ratio, and packing efficiency which are all potential regulators of crucial nuclear processes^42,46^ can be characterized to understand the implications of chromatin structure on gene expression and vice-versa. Future experiments are necessary to elucidate the molecular basis of these packing domains as it is currently unknown whether packing domains observed with ChromSTEM are the same as other domain-like structures of similar average sizes that are observable with different methodologies.

Our previous experiments on isogenic cell lines have demonstrated *D* as a crucial modulator of transcriptional plasticity^46^. In this study, we obtained the distribution of packing scaling D, with median values and lower and upper quartiles of $2.64$ (Q_1_ = 2.53, Q_3_ = 2.73) for A549 cells (Fig. 4A), and $2.63$ (Q_1_ = 2.53, Q_3_ = 2.71) for BJ cells (Fig. S7C). This given range in *D* values within the same cell line implies that genes may be localized into packing domains with different *D* values depending on how responsive the gene must be to external stimuli and that this could be potentially co-opted in the cancer cell state for chemoevasion purposes. Furthermore, for packing domains of both A549 and BJ cell lines, we observed a diverse range of average chromatin densities, domain sizes, asphericities, and exposure ratios, all of which could also impact transcription rate (Fig. 4). As domains adopt a packing structure with similar mass scaling behavior, some of these morphological properties are interrelated from a polymer physics perspective. From ChromSTEM data, we observed chromatin density and packing scaling cannot be related by a universal factor, and this packing efficiency varies between domains within the same cell line. We also observed a similarly complex relationship between the exposure ratio, the probability of a chromatin segment being on the domain surface, and domain size. The heterogenous morphological properties of domains could potentially play a role in regulating gene activities by controlling the size of proteins and other macromolecular complexes that can navigate through this network, thus influencing material transportation and gene accessibility.

The major limitations of ChromSTEM/TEM include chemical fixation, low throughput due to electron tomography, and the inability to obtain locus-specific information. Therefore, ChromSTEM findings are not directly comparable to discoveries made from sequencing-based techniques such as Hi-C or locus-based imaging methods such as Fluorescence In Situ Hybridization (FISH). Additionally, ChromSTEM involves reagents such as DRAQ5, DAB, and osmium for DNA-specific labeling that may alter nucleotide structure. The reagents are added after chemical fixation to minimize the effect. Dehydration and resin embedding are also known to create unavoidable volume changes. In our sample preparation protocol, Durcupan was used because it has been reported as one of the most stable resins that minimized sample shrinkages^68^.

Despite its limitations, we believe that ChromSTEM and the associated analysis methods developed in this work should become an important tool for understanding the 3D structure and function of chromatin. Additionally, we have recently demonstrated that results from high-resolution ChromSTEM imaging can be combined with other modalities in our Nanoscale Chromatin Imaging and Analysis (nano-ChIA) platform to quantify different aspects of the chromatin structure which provide information about molecular functionality and high-throughput chromatin dynamics imaging in live cells^42^.

Our computational model of transcription allows us to understand the functional consequences of changes in packing domain size (Rf), average chromatin density (CVC), and chromatin packing scaling (*D*), which might be distinct for heterochromatic versus euchromatic domains. Previous work has demonstrated that heterochromatin is packed into larger and higher CVC domains. However, the exact relationship between heterochromatin epigenetic modifiers and chromatin packing scaling has not been irrefutably determined. The transcription model predicts that *D* controls the variance of local crowding conditions that genes in a specific domain are exposed to as well as accessible surface to transcriptional reactants, including RNA pol II. Thus, changes in *D* could modulate the transcriptional activity of genes within a heterochromatic domain and future work should focus on characterizing the relationship between *D* and epigenetic states across multiple cell lines. As a preliminary study, we utilized ChromTEM to estimate differences in *D* based on the spatial location of chromatin within the nucleus. Chromatin at the nuclear periphery is highly dense, presumably rich in heterochromatin-specific histone modifications like H3K9me2/3^60^, and is constrained by interactions with the proteins at the inner nuclear membrane-such as lamins and HP1^69^. Analysis of local chromatin motion within the whole nucleus has revealed that chromatin at the nuclear periphery is less mobile due to the topological constrains^69^. Our work demonstrates that the high-density chromatin at the nuclear periphery has higher *D* compared to the chromatin in the interior regions (Fig. 5).

In the future, co-registering ChromSTEM with 3D super-resolution techniques which enables labeling of markers for heterochromatin and euchromatin^31^ will be integral to improving our understanding of the relationships between the physical structure of chromatin within packing domains, epigenetic modifications, and transcription. Future work should also focus on developing novel locus-specific labeling methods that are compatible with ChromSTEM sample preparation and imaging, and colocalizing chromatin morphological and genetic information for a greater number of cells. For example, labeling TADs identified by Hi-C experiments on the same cell lines and co-registering them with ChromSTEM could help to elucidate the relationship between packing domains and TADs. Additionally, such studies could help to uncover a domain-specific relationship between contact probability scaling and chromatin packing scaling, which has been investigated in previous studies for simpler polymer models^42^. Altogether, these experiments would help to better understand the functional consequences of heterogeneous packing domain organization.

## Materials and methods

### Cell culture

A549 and BJ cell lines were purchased from American Type Culture Collection (ATCC Manassas, VA). A549 cells were cultured in Dulbecco's Modified Eagle Medium (ThermoFisher Scientific, Waltham, MA, #11965092). BJ cells were cultured in Minimum Essential Media (ThermoFisher Scientific, Waltham, MA, #11095080). All cells were maintained at physiological conditions (5% CO_2_ and 37 °C). Experiments were performed on cells from passages 5–20.

### ChromEM sample preparation

The ChromSTEM sample staining and resin-embedding followed the published protocol^15^, and detailed reagents can be found in Table S1. All cells were thoroughly rinsed in Hank’s balanced salt solution without calcium and magnesium (EMS) before fixation with EM fixative. Two stages of fixation were performed: room temperature fixation for 5 min and on-ice fixation for an hour with fresh fixative. The cells were kept cold for all following steps before resin embedding either on ice or a cold stage with the temperature monitored to vary from 4 to 10 °C. After fixation, the samples were bathed in the blocking buffer for 15 min before being stained by DRAQ5™ (Thermo Fisher) for 10 min. The cells were rinsed and kept in the blocking buffer before photo-bleaching and submerged in 3–5′-diaminobenzidine (DAB) solution (Sigma Aldrich) during photo-bleaching on the cold stage.

A Nikon microscope (Nikon Inc.) was used for photo-bleaching. A cold stage was developed in-house from a wet chamber equipped with humidity and temperature control. After photo-bleaching, the cells were rinsed in 0.1 M sodium cacodylate buffer thoroughly. Reduced osmium solution (EMS) was used to enhance the contrast in STEM HAADF mode, and the heavy metal staining lasted 30 min on ice. Serial ethanol dehydration was performed, and during the last 100% ethanol wash, the cells were brought back to room temperature. Durcupan resin (EMS) was used for embedding after infiltration, and the blocks were cured at 60 °C for 48 h.

An ultramicrotome (UC7, Leica) and a 35-degree diamond knife (DiATOME) were employed to prepare sections of different thicknesses. For STEM HAADF tomography, semi-thick sections were made and deposited onto a copper slot grid with carbon/Formvar film. All TEM grids were plasma cleaned before sectioning and no post-staining was performed on the sections. 10 nm colloidal gold fiducial markers were deposited on both sides of the sample.

A step-by-step protocol can be found in Protocol S1.

### EM data collection and tomography reconstruction

A 200 kV cFEG STEM (HD2300, HITACHI) with HAADF mode was employed for all image collection. For tomography, the sample was tilted from − 60° to 60° with 2° increments on two roughly perpendicular axes. Each tilt series was aligned with fiducial markers in IMOD^53^ and reconstructed using Tomopy^54^ with a penalized maximum likelihood for 40 iterations independently. IMOD^53^ was used to combine the tomograms to suppress artifacts (Fig. S2)^55^. The voxel size varies from 1.8 to 2.9 nm for different samples. Therefore, based on the Nyquist criterion, the resolution is between 4 and 6 nm for the same samples. Volume Viewer in FIJI was employed for surface rendering^56^. In summary, we performed dual-tilt tomography reconstruction in combination with a penalized maximum likelihood reconstruction algorithm to suppress artifacts associated with “missing wedge”. To avoid the direct influence of elongation artifacts along the z-axis, we also evaluated mass scaling and domain morphological properties in the x–y plane and averaged them along the z-axis.

### Chromatin mass scaling analysis

For a polymer with power-law mass scaling behavior, the mass scales as $\left(r\right)\propto {r}^{D}$, where $D$ is the power-scaling exponent. To calculate mass scaling, starting from the ChromSTEM image where intensity is linearly related to mass, with concentric circles of increasing radius starting at all centers selected within the sample, *M* is calculated as the total mass of voxels weighted by the intensity value at the center within the predefined space (perimeter, area, and volume). For each stack of tomography, we averaged mass scaling curves with different centers at each dimension. For A549 cells, 1D, 2D, and 3D mass scaling were analyzed. For BJ cells, only 1D and 2D mass scaling were evaluated, as the thickness of the tomography is limited. We found from our calculations from polymer trajectories that the 3D mass scaling exponent can be approximated using the 2D case and the 1D case (Fig. S4A): ${D}_{3D}={D}_{2D}+1$, and ${D}_{3D}={D}_{1D}+2,$ with standard errors of the mean of 0.023 and 0.019 respectively.

### Domain center identification and boundary estimation analysis

For each z-stack of tomography, we first evaluated the grayscale chromatin mass density projection (Fig. S5). Then we applied Gaussian filtering with radius = 5 pixels followed by CLAHE contrast enhancement with a block size of 120 pixels in FIJI. We then identified the local maxima from the projection images to identify chromatin domains with enhanced contrast in FIJI. Next, we identified the center pixel of gravity per segmented domain. To obtain the mass scaling curve for a single domain, we first sampled multiple mass scaling curves with centers on the nonzero pixels around the identified domain center within an 11-pixel × 11-pixel window. We then used the average mass scaling curve for that domain for subsequent analysis.

To obtain the domain boundary, we utilized the mass scaling behavior of the packing from the center region to the periphery. To evaluate such behavior, besides the mass scaling curve, we also leveraged the radial volume chromatin concentration (CVC). We adopt the definition of CVC from published work^15^: which is the fraction of volume occupied by chromatin. The boundary of the domain can be seen as the length scale where a single power-law relationship no longer holds is defined as the domain size *R*_*f*_. Practically, we used the smallest length scale that meets at least one of the three criteria (Fig. S6): 1. Mass scaling curve deviates from the initial power-law calculated from small length scales by 5%, suggesting a significantly different packing behavior; 2. Local packing scaling *D* reaches 3 (supra-domain regime); 3. The radial CVC starts to increase. Similar to *R*_*f*_, the average size of the fundamental building block of the domain *R*_*min*_ can be measured by the spatial separation where the mass scaling behavior deviates significantly (5%) from the behavior within the initial chromatin fiber regime. The ratio between *R*_*f*_ and *R*_*min*_ is defined as the effective domain size *R*_*eff*_.

### Domain morphological property analysis

We calculated four different morphological properties for each domain: packing scaling *D*, asphericity *A*_*s*_, domain CVC, and exposure ratio (ER). The distributions in Fig. 4, S6, and S7 are shown as mean ± S.D. using violin super plots^70^. Interquartile range or IQR is defined as the range in terms of the first quartile, Q_1,_ and the third quartile, Q_3_. Chromatin packing scaling as *D*_*log*_ was estimated by first estimating the linear regions from the average 2D mass scaling curves in log–log and then finding the slope of the linear regression of the average mass scaling curves. Chromatin within *R*_*f*_ distance from the domain center pixel was selected as a “domain”, though realistically the domains are likely to adopt an irregular shape. The 2D asphericity *A*_*s*_ is calculated slice-by-slice using the following expression: ${A}_{s}=\frac{{({{\lambda }_{1}}^{2}-{{\lambda }_{2}}^{2})}^{2}}{{({{\lambda }_{1}}^{2}+{{\lambda }_{2}}^{2})}^{2}}$, where ${\lambda }_{1}$ and ${\lambda }_{2}$ are the eigenvalues of the 2D gyration tensor of the domain^63^. We then calculated the mean value from each slice to be the *A*_*s*_ for that domain. The CVC is calculated as the ratio of the total number of nonzero (chromatin) voxels over the total number of voxels per domain. And the exposure ratio is the fraction of voxels on the domain surface. The surface of the domain includes only the surface of the pores within the domain; it excludes the external surface created by an artificial boundary imposed by *R*_*f*_.

### ChromTEM—ACF analysis for nuclear interior and periphery

Analysis for chromatin at the nuclear periphery and the interior was performed using ChromTEM. Following ChromEM sample preparation, TEM images of 50-nm-thin sections of BJ cells were analyzed. For the thin sections, a TEM (HT7700, HITACHI) was operated at 80 kV in the bright field to capture high-contrast chromatin data. From the radial chromatin density as a function of distance from the nuclear periphery (Fig. 5A), we first estimated the average thickness of chromatin at the nuclear periphery to be about 300 nm. This is because for distances up to 300 nm from the nuclear periphery, the chromatin density is relatively high and at distances greater than ~ 300 nm the chromatin density starts decreasing gradually. Each nucleus, 300 nm thick periphery from the nuclear boundary, and non-peripheral region were carefully segmented in FIJI^56^, and nucleoli and any sectioning artifacts were excluded from the analysis. The chromatin packing scaling, *D* was calculated from the spatial autocorrelation function of chromatin density variations, ACF for these regions within the nucleus as previously described^42^. The local chromatin packing *D*_*log*_ was estimated from the linear regression on ACF in a log–log scale for 25 nm < *r* < 60 nm for chromatin at the periphery, interior, and whole nuclei.

## Supplementary Information

Supplementary Information 1.

Supplementary Video 1.

Supplementary Video 2.

Supplementary Video 3.

Supplementary Video 4.

Supplementary Video 5.

Supplementary Video 6.

Supplementary Video 7.

Supplementary Video 8.

Supplementary Video 9.

## Data Availability

The raw datasets generated during and/or analyzed during the study are available from the corresponding author on reasonable request.
