# Author Correction: Analysis of three-dimensional chromatin packing domains by chromatin scanning transmission electron microscopy (ChromSTEM)

**DOI:** 10.1038/s41598-022-17293-x

**Published:** 2022-07-26

**Authors:** Yue Li, Vasundhara Agrawal, Ranya K. A. Virk, Eric Roth, Wing Shun Li, Adam Eshein, Jane Frederick, Kai Huang, Luay Almassalha, Reiner Bleher, Marcelo A. Carignano, Igal Szleifer, Vinayak P. Dravid, Vadim Backman

**Affiliations:** 1 Applied Physics Program, Northwestern University, Evanston, IL 60208 USA; 2 Department of Biomedical Engineering, Northwestern University, Evanston, IL 60208 USA; 3 Department of Materials Sciences and Engineering, Northwestern University, Evanston, IL 60208 USA; 4 Shenzhen Bay Laboratory, Institute of Systems and Physical Biology, Shenzhen, 518132 China; 5 Medical Scientist Training Program, Feinberg School of Medicine, Northwestern University, Evanston, IL 60611 USA; 6 Department of Chemistry, Northwestern University, Evanston, IL 60208 USA

Correction to: *Scientific Reports* 10.1038/s41598-022-16028-2, published online 16 July 2022

The Acknowledgments section in the original version of this Article was incomplete.

“This work was supported by Medical Scientist Training Program T32GM008152, National Institutes of Health grants U54CA193419, R01 CA228272, R01CA225002, and National Science Foundation grants EFMA-1830961 and EFMA-1830969. This work made use of the BioCryo facility of Northwestern University’s NUANCE Center, which has received support from the SHyNE Resource (NSF ECCS-2025633), the International Institute for Nanotechnology (IIN), and Northwestern's MRSEC program (NSF DMR-1720139). The imaging methodology is partially based on research sponsored by the Air Force Research laboratory under agreement number FA8650-15-2-5518. The U.S. Government is authorized to reproduce and distribute reprints for Governmental purposes notwithstanding any copyright notation thereon. The views and conclusions contained herein are those of the authors and should not be interpreted as necessarily representing the official policies or endorsements, either expressed or implied, of Air Force Research Laboratory or the U.S. Government.”

now reads:

“This work was supported by Medical Scientist Training Program T32GM008152, National Institutes of Health grants U54CA268084, U54CA261694, R01CA228272, R01CA225002, and National Science Foundation grants EFMA-1830961 and EFMA-1830969. This work made use of the BioCryo facility of Northwestern University’s NUANCE Center, which has received support from the SHyNE Resource (NSF ECCS-2025633), the International Institute for Nanotechnology (IIN), and Northwestern's MRSEC program (NSF DMR-1720139). The imaging methodology is partially based on research sponsored by the Air Force Research laboratory under agreement number FA8650-15-2-5518. The U.S. Government is authorized to reproduce and distribute reprints for Governmental purposes notwithstanding any copyright notation thereon. The views and conclusions contained herein are those of the authors and should not be interpreted as necessarily representing the official policies or endorsements, either expressed or implied, of Air Force Research Laboratory or the U.S. Government.”

The original Article has been corrected.

