# Supplementary material for: Analysis of three-dimensional chromatin packing domains by chromatin scanning transmission electron microscopy (ChromSTEM)
Source: Sci Rep. 2022 Jul 16;12:12198. doi: 10.1038/s41598-022-16028-2 (PMC9288481; doi:10.1038/s41598-022-16028-2)
Supplement: Supplementary file 1 — Supplementary Information 1. [file 41598_2022_16028_MOESM1_ESM.docx]

**Analysis of Three-Dimensional Chromatin Packing Domains by Chromatin Scanning Transmission Electron Microscopy (ChromSTEM)**

Yue Li^1,+^, Vasundhara Agrawal^2,+^, Ranya K. A. Virk^2,+^, Eric Roth^3^, Wing Shun Li^1^, Adam Eshein^2^, Jane Frederick^2^, Kai Huang^4^, Luay Almassalha^5^, Reiner Bleher^3^,

^1^ Applied Physics Program, Northwestern University, Evanston, Illinois 60208, USA.

^2^ Department of Biomedical Engineering, Northwestern University, Evanston, Illinois 60208, USA.

^3^ Department of Materials Sciences and Engineering, Northwestern University, Evanston, Illinois 60208, USA.

^4^ Institute of Systems and Physical Biology, Shenzhen Bay Laboratory, Shenzhen 518132, China

^5^ Medical Scientist Training Program, Feinberg School of Medicine, Northwestern University, Evanston, Illinois 60611, USA.

^6^ Department of Chemistry, Northwestern University, Evanston, Illinois 60208, USA.

^+^ These authors contributed equally to the manuscript.

^*^Correspondence and requests for materials should be addressed to V.B. (email: [v-backman@northwestern.edu](mailto:v-backman@northwestern.edu)) or V.P.D. (email: [v-dravid@northwestern.edu](mailto:v-dravid@northwestern.edu))

**Supporting Information**

**Mov. S1.** Tomogram of the 3D chromatin structure moving through the z-direction of the chromatin in the A549 cell in Fig. 1

**Mov. S2.** Tomogram of the 3D chromatin structure moving through the z-direction of the chromatin in the BJ cell in Fig. S1

**Mov. S3.** Tomogram of the 3D chromatin structure moving through the z-direction of the chromatin in another A549 cell

**Mov. S4.** 3D rendering of the entire tomography stack of **Mov. S1**

**Mov. S5.** 3D rendering of nucleosomal superstructures from the same A549 cell as in **Mov. S1**

**Mov. S6.** 3D rendering of the entire tomography stack of **Mov. S2**

**Mov. S7.** 3D rendering of nucleosomal superstructures from the same BJ cell as in **Mov. S2**

**Mov. S8.** 3D rendering of the entire tomography stack of **Mov. S3**

**Mov. S9.** 3D rendering of nucleosomal superstructures from the same A549 cell as in **Mov. S3**

**Protocol S1. Sample preparation for ChromSTEM for cell cultures**

**Fixation:**

1. Wash the cells in the petri-dish in the washing solution for 3 times, 2 minutes each.
2. Fix the cells with the fixation solution for 5 minutes at room temperature.
3. Continue to fix the cells with fresh fixation solution for an additional 1 hour on ice.

The following steps before the last ethanol dehydration are either on ice or a cold stage, all reagents must be chilled to 4^o^C before use.

**DNA Staining:**

1. Wash the cells with 0.1M sodium cacodylate buffer for 5 times on the ice, 2 minutes each.
2. Block the cells with a blocking solution for 15 minutes.
3. Stain the cells with DNA staining solution for 10 minutes.
4. Wash the cells with the blocking solution 3 times, 5 minutes each.

**Photo-bleaching:**

1. Bath the cells in the bathing solution before photo-bleaching
2. Photo-bleach the cells using continuous epi-fluorescence illumination (150 W Xenon Lamp) with Cy5 red tilter and a 100x objective for 7 minutes for each spot on the cold stage.
3. Replace the bathing solution in the petri-dish with a fresh bathing solution every 15 minutes (roughly two spots).

**Heavy metal staining:**

1. Rinse the cells with 0.1 M sodium cacodylate buffer 5 times, 2 minutes each.
2. Stain the cells with a reduced osmium staining solution for 30 minutes.
3. Wash the cells with double distilled water 5 times, 2 minutes each.

**Dehydration and Resin embedding:**

1. Dehydrate the cells with serial ethanol (30%, 50%, 70%, 85%, 95%, 100% twice) on ice, 2 minutes each.
2. Wash the cells with 100% ethanol at room temperature for 2 minutes.
3. Infiltrate the cells with a 1:1 infiltration mixture at room temperature for 30 minutes.
4. Infiltrate the cells with a 2:1 infiltration mixture at room temperature for 2 hours.
5. Infiltrate the cells with Durcupan ^TM^ resin mixture 1 at room temperature for 1 hour.
6. Infiltrate the cells with Durcupan ^TM^ resin mixture 2 at 50^o^C in the dry oven for 1 hour.

Flat embed the cells with fresh Durcupan ^TM^ resin mixture 2 in Beem capsule and cure at 60 ^o^C in the dry oven for 48 hours.

**Table S1. Reagents used in ChromSTEM Staining**

| **Reagent** | **Formula** |
| --- | --- |
| Washing solution | Hank’s balanced salt solution without calcium and magnesium |
| Fixation solution | 2.5% EM grade glutaraldehyde  2% paraformaldehyde  2 mM CaCl_2_  0.1 M sodium cacodylate buffer, pH = 7.4 |
| Blocking solution | 10 mM glycine  10 mM potassium cyanide  0.1 M sodium cacodylate buffer, pH = 7.4 |
| DNA staining solution | 10 µM DRAQ5  0.1% SAPONIN  0.1 M sodium cacodylate buffer, pH = 7.4 |
| Bathing solution | 2.5 mM 3,3’- diaminobenzidine tetrahydrochloride (DAB)  0.1 M sodium cacodylate buffer, pH = 7.4 |
| Reduced osmium staining solution | 2% osmium tetroxide  1.5% potassium ferrocyanide  2 mM CaCl_2_  0.15 M sodium cacodylate buffer, pH = 7.4 |
| Durcupan ^TM^ resin mixture 1 | 10 mL Durcupan ^TM^ ACM single component A, M, epoxy resin  10 mL Durcupan ^TM^ ACM single component B, hardener 964  0.15 mL Durcupan ^TM^ ACM single component D |
| Durcupan ^TM^ resin mixture 2 | 10 mL Durcupan ^TM^ ACM single component A, M, epoxy resin  10 mL Durcupan ^TM^ ACM single component B, hardener 964  0.2 mL Durcupan ^TM^ ACM, single component C, accelerator 960  0.15 mL Durcupan ^TM^ ACM single component D |
| 1:1 infiltration mixture | 10 mL 100% ethanol  10 mL Durcupan ^TM^ resin mixture 1 |
| 2:1 infiltration mixture | 5 mL 100% ethanol  10 mL Durcupan ^TM^ resin mixture 1 |


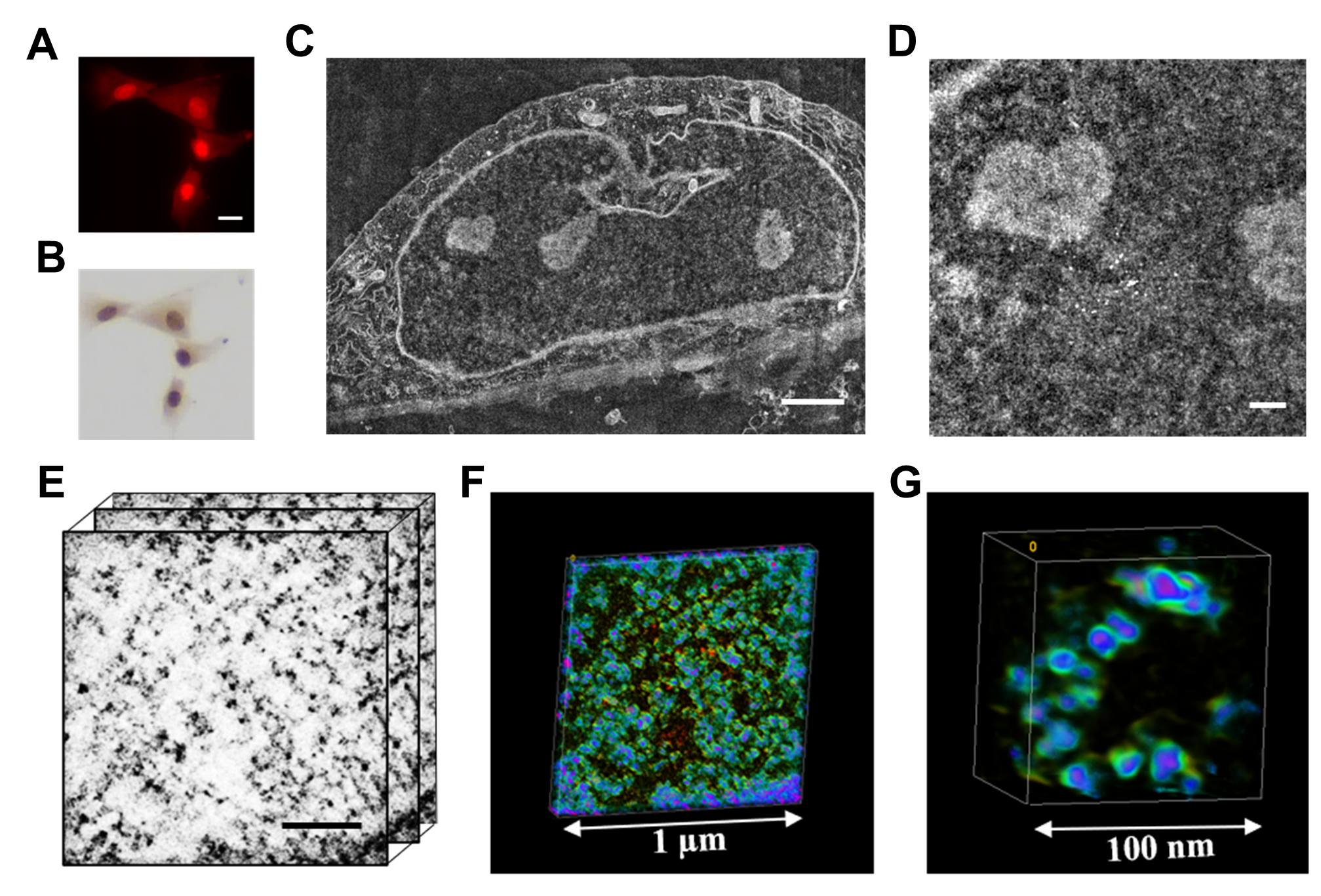


**Fig. S1.** **ChromSTEM tomography reconstruction of the chromatin of a BJ cell.** (**A**) Photo-oxidation by DRAQ5, fluorescence image of a group of four BJ cells. Scale bar: 20 µm (**B**) Same group of cells after resin embedding show higher contrast under a bright-field optical microscope. (**C**) HAADF image of a BJ cell nucleus prepared with ChromEM staining method. Scale bar: 2 µm. (**D**) HAADF image at higher magnification of the same BJ cell nucleus in (**C**), the colloidal gold nanoparticles are visible and used as fiducial markers in tomography collection. Scale bar: 500 nm. (**E**) A stack of virtual 2D tomograms of the chromatin of the same BJ cell. Scale bar: 200 nm. (**F**) 3D volume rendering, color-coded by the tomogram voxel intensity. The tomogram voxel intensity increases from green to blue to pink. (**G**) Nucleosomal superstructures with higher voxel intensity are identifiable at higher magnification.


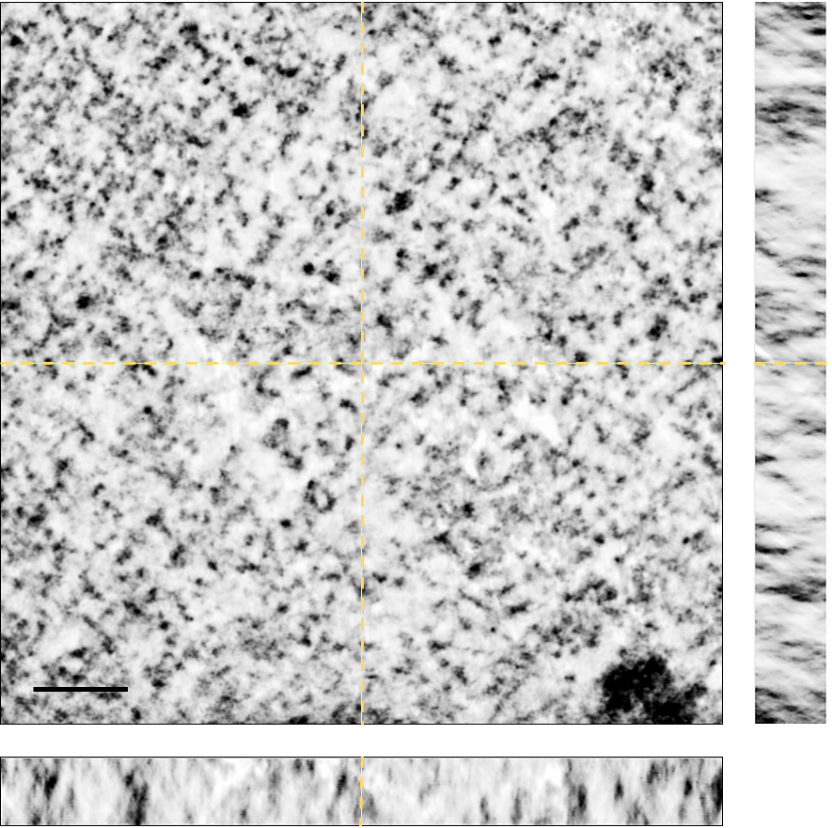


**Fig. S2. Orthogonal view of the reconstructed tomography for A549 nucleus.** To suppress artifacts associated with “missing wedge”, we performed dual-tilt tomography reconstruction in combination with a penalized maximum likelihood reconstruction algorithm. In the side view (x-z and y-z directions) there are still streaks visible due to the “missing cone”, but the contrast of nucleosomes is significantly higher than the noise and sufficient for chromatin mass density analysis. Scale bar: 200 nm.

**
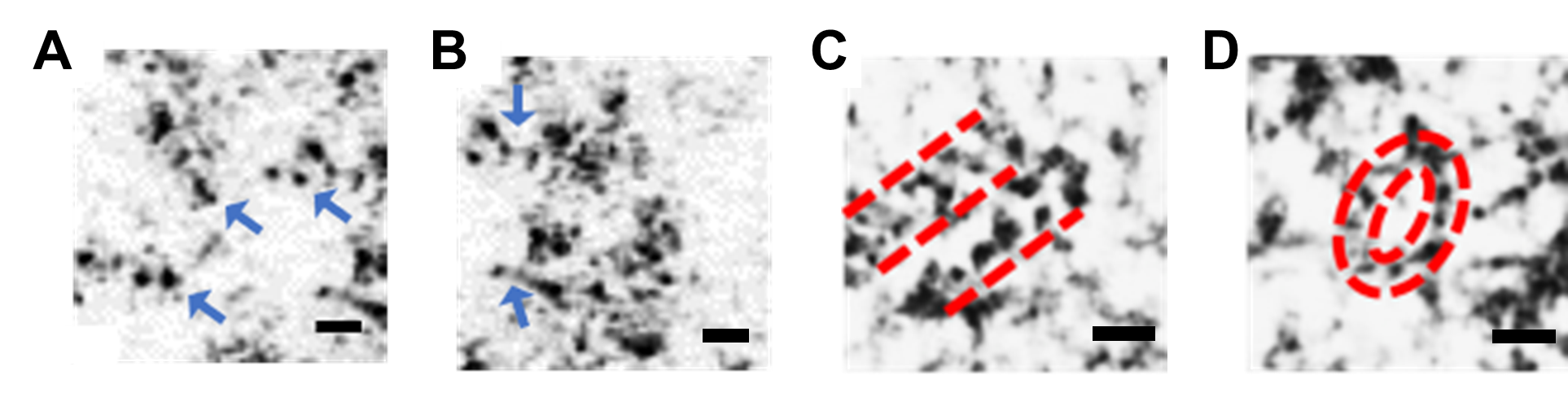
**

**Fig. S3. ChromSTEM resolves distinct higher-order supranucleosomal structures.** Magnified views of the tomograms from BJ cell nuclei (Fig. S1E) with details of nucleosomes (blue arrows in **A**) and linker DNA between them (blue arrows in **B**). Scale bar: 30 nm. Rare chromatin conformations such as supranucleosomal stacking (red dashed lines in **C**), and rings (red dashed circles in **D**) were observed in A549 cell nuclei (Fig.1F). Scale bar: 30 nm.

**
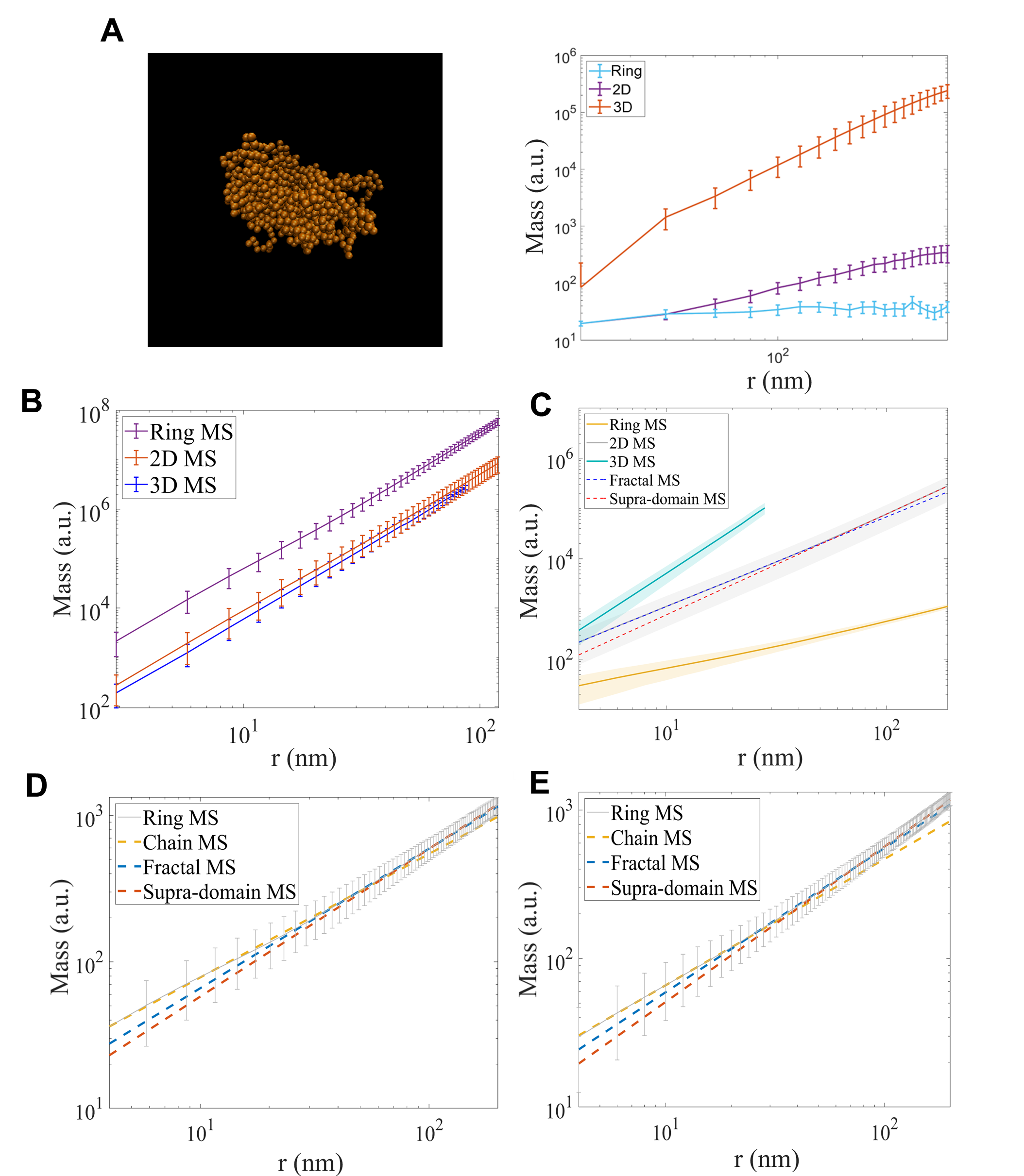
Fig. S4. Mass scaling analysis at different dimensions. (A)** Rendering of a self-attracting homopolymer with D = 2.37 (left) which was estimated from 3D, 2D, and Ring Mass scaling (right) by performing linear regression in the log-log scale on the mass scaling curves for the given dimensions within 20 nm - 240 nm. 3D mass scaling exponent can be approximated using the 2D case and the 1D case: $D_{3D}=D_{2D}+1$ and $D_{3D}=D_{1D}+2,$ with standard errors of the mean of 0.023 and 0.019 respectively. **(B)** Mass scaling curves for A549 cells were plotted for different dimensions as 3D MS, 2D MS + *r*, and Ring MS + *2r* in the log-log scale. The equivalent slope for the 3D mass scaling regime extending from 2 nm to ~100 nm indicates that the 3D mass scaling exponent can be derived from 2D and ring mass scaling exponents. (**C**) The average mass scaling (MS) curves were determined from different dimensions of the amalgam of ChromSTEM tomograms from three BJ cells. In the 2D cases for BJ cells, the MS curve starts with a packing scaling *D_log_* < 3 (blue dashed line) and smoothly transitions to values close to *D_log_* = 3 (red dashed line). (**D**) The ring mass scaling curve for A549 cells seems to show three regimes: 1. Chain MS with slope, $D=2.84\pm0.05$ fitted from *r* = 2 nm to 14.5 nm (yellow dashed line); 2. Domain MS (blue dashed line); 3. Supra-domain MS with slope*,* $D=3.01\pm0.01$ fitted from *r* = 145 nm to 200 nm (red dashed line). (**E**) The ring mass scaling curve for BJ cells shows similar three power-law regimes: 1. Chain MS with slope, $D=2.85\pm0.03$ fitted from *r* = 2 nm to 10 nm (yellow dashed line); 2. Domain MS (blue dashed line) ; 3. Random MS with slope, $D=3.04\pm0.003$ fitted from *r* = 100 nm to 140 nm (red dashed line).


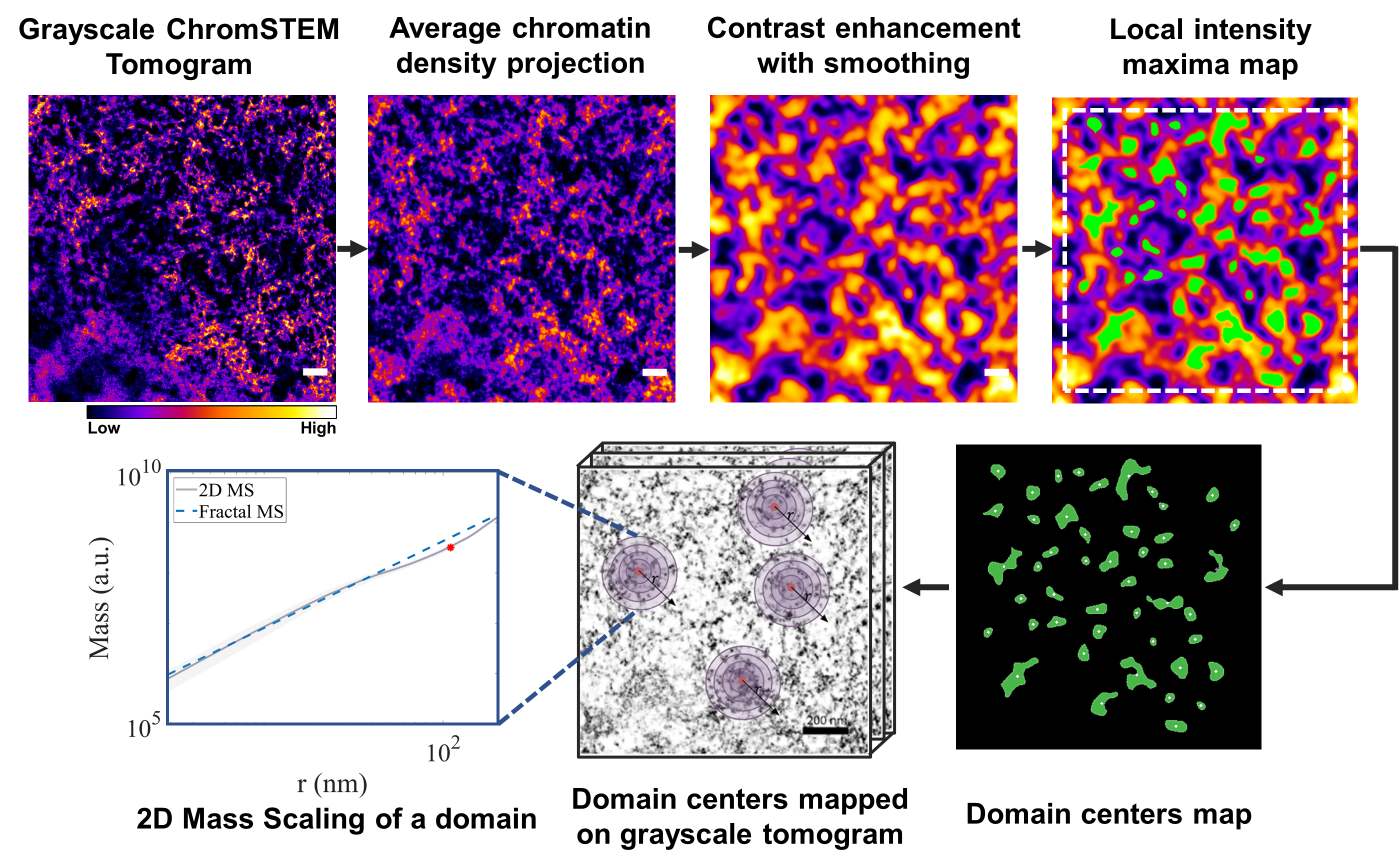


**Fig. S5. Chromatin density analysis to identify chromatin domain centers.** The average z-projection for the grayscale tomograms was evaluated to obtain a map of the average chromatin density distribution using ChromSTEM-HAADF intensity. Then we applied Gaussian filtering with radius = 5 pixels followed by CLAHE contrast enhancement with a block size of 120 pixels in FIJI. We identified the local maxima for unbiased segmentation of chromatin domains. We then identified the center of mass pixel for each segmented domain. To obtain the mass scaling curve for a single domain, we first sampled multiple mass scaling curves starting from the nonzero pixels within the domain centers, defined as the 11-pixel x 11-pixel window surrounding the center pixel. We then used the average mass scaling curve for that domain for subsequent analysis. Scale bar: 200 nm.

**
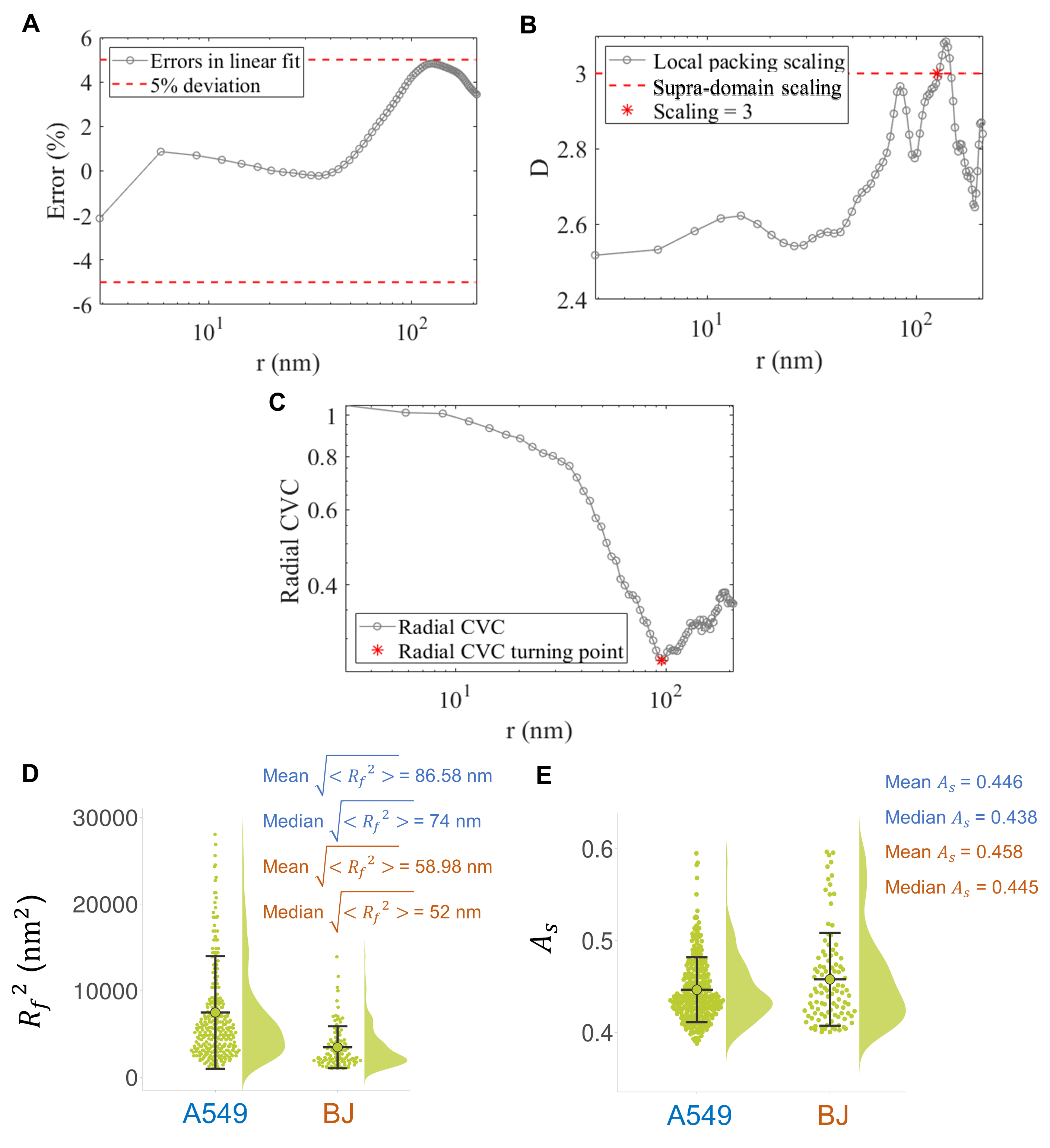
**

**Fig. S6. Determining domain boundaries from the mass scaling and radial density behavior**. Beyond a given length scale, *r* (nm), the 2D mass scaling curve for a given domain deviates from a power-law mass scaling. We performed three types of analyses (**A-C**) to determine the boundary of individual domains, which are denoted as the smallest r of the three analyses if it exists. (**A**) The mass scaling curve deviates from the initial power-law mass scaling calculated from small length scales within the domain center region by 5%. (**B**) Local packing scaling $D_{log}$ reaches 3, which can be denoted as the supra-domain scaling regime. Here, the packing scaling = 3 at *r* = 102 nm. **C**) The radial CVC starts to increase. The radial CVC decreases initially, then increases at *r* = 95.7 nm for this given domain. In this case, comparing (**A**-**C**), we determined the size of this domain to be *R_f_* = 95.7 nm. (**D**) The distribution of *R_f_^2^*, the square of the radius of the packing domain, for A549 (blue) and BJ (orange) cells. (**E**) The distribution of *A_s_*, the asphericity of the chromatin density distribution within the identified domains, for A549 (blue) and BJ (orange) cells. 280 and 140 packing domains were analyzed for A549 and BJ cells respectively.


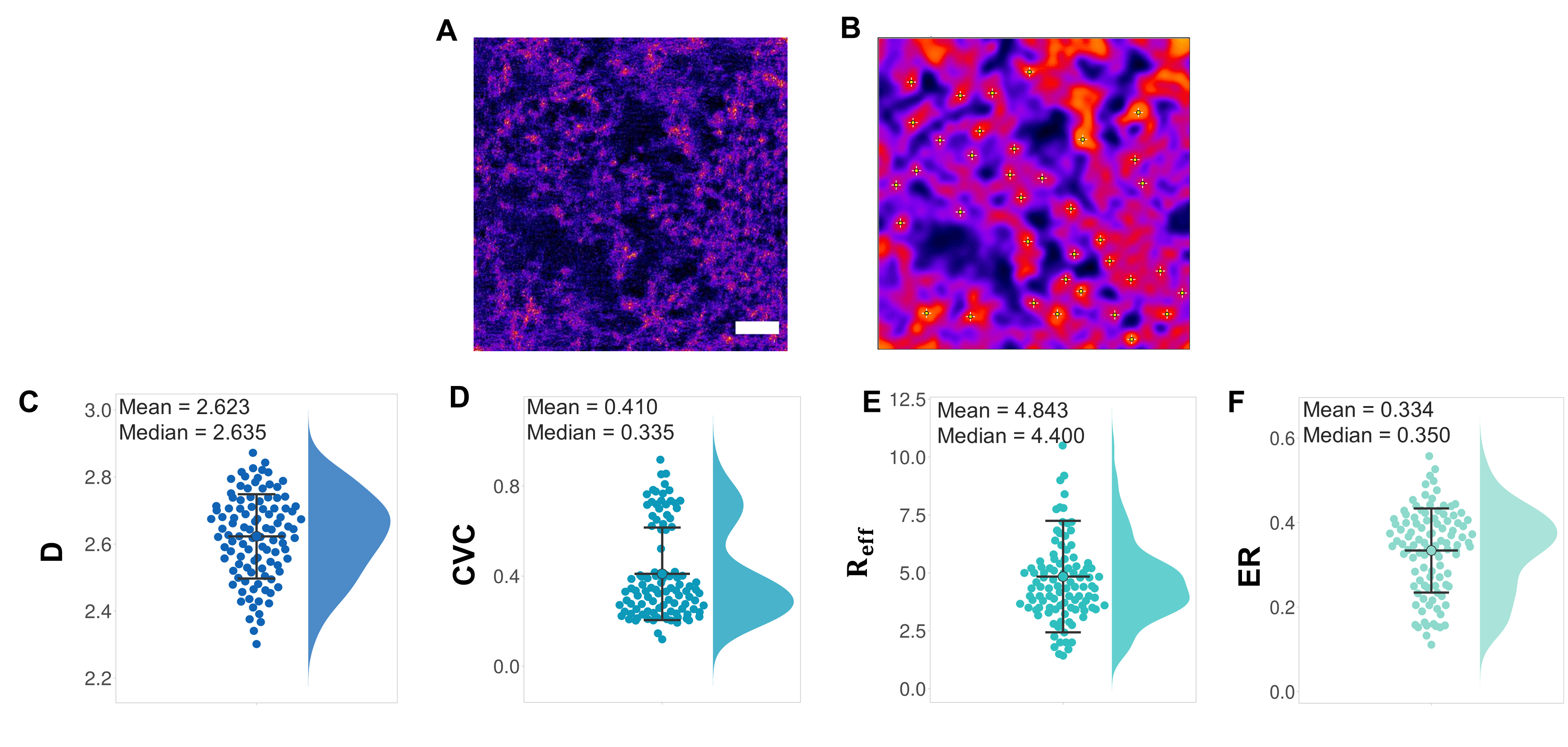


**Fig. S7. Characterizing morphological properties of chromatin packing domains in BJ cells.** The grayscale BJ cell tomogram **(A)** was utilized to estimate the **(B)** chromatin domain centers. Scale bar: 100 nm. **(C)** Chromatin packing scaling *D* distribution with a median (IQR) equal to 2.63 (2.53-2.71) was evaluated for BJ cells. **(D)** CVC distribution ranges from 12% to 92% with a median value of 34%. **(E)** The median effective domain size *R_eff_* was 4.40 (3.5-5.4), and the median *R_min_* is 10.8 (10-14.4) nm. **(F)** The Exposure Ratio, *ER* ranges from 0.11 to 0.56 with a median value of 0.35.
